# Plant-Mediated Silver Nanoparticle Formulations for Wound Healing: A Process–Property–Performance Perspective

**DOI:** 10.3390/pharmaceutics18091161

**Published:** 2026-09-15

**Authors:** Mohammad Moein Sadeghi, Souha Idoudi, Helena M. Kelly, Nashiru Billa

**Affiliations:** 1 Department of Pharmaceutical Sciences, College of Pharmacy, QU Health, Qatar University, Doha P.O. Box 2713, Qatar; m.moein@qu.edu.qa (M.M.S.); si1906739@qu.edu.qa (S.I.); 2 School of Pharmacy & Biomolecular Sciences, Royal College of Surgeons in Ireland (RCSI), D02 VN51 Dublin, Ireland; helenakelly@rcsi.ie

**Keywords:** silver nanoparticles, AgNPs, green synthesis, plant-mediated synthesis, wound healing, wound dressing

## Abstract

Biomaterials intended for wound healing are expected to reduce microbial load while supporting tissue repair in complex wound environments. Plant-mediated synthesis of silver nanoparticles (AgNPs) has attracted interest because plant extracts can provide phytochemicals that mediate nanoparticle reduction, capping, and stabilization. This review critically evaluates recent studies on plant-mediated AgNPs for wound-healing applications, focusing on botanical and extraction variables, synthesis conditions, physicochemical properties, formulation strategies, administered exposure, safety, and biological outcomes. Across the 34 included publications, 14 directly assessed a process-to-property relationship and only two assessed a process-to-performance relationship; none directly assessed a property-to-performance relationship or established a complete process-to-property-to-performance chain. The reviewed studies suggest that plant-mediated AgNPs may reduce microbial burden, modulate inflammation, mitigate oxidative stress, and promote cell migration, re-epithelialization, collagen remodeling, and angiogenesis. However, batch-to-batch variability, incomplete reporting of experimental details, over-reliance on ultraviolet–visible (UV–Vis) spectroscopy for optimization, inadequate evaluation of formulation stability, inconsistent dose reporting, and limited molecular validation of proposed mechanisms remain major factors limiting translation. By evaluating plant-mediated AgNP-based wound-healing systems through a process–property–performance framework, this review summarizes recent advances and highlights key considerations for improving reproducibility, comparability, and translational relevance.

## 1. Introduction

Wound healing is a complex process involving multiple overlapping stages, including hemostasis, inflammation, proliferation, re-epithelialization, angiogenesis, extracellular matrix deposition, and remodeling [1]. While acute wounds generally heal in a biologically programmed and timely manner, wound healing may be delayed or dysregulated under various clinical conditions, including burns, traumatic and surgical wounds, pressure ulcers, venous ulcers, and diabetic wounds. Chronic and nonhealing wounds represent a significant clinical and socioeconomic burden [2]. Chronic wounds represent a significant public health concern, affecting one in six Medicare beneficiaries in the United States, or approximately 10.5 million individuals. Annual Medicare expenditures associated with chronic wound care have been estimated at $22.5 billion in the United States. Global wound-care expenditure was estimated at $148.65 billion in 2022 [3]. Delayed wound repair can result from several factors, including prolonged inflammation, oxidative stress, poor vascularization, impaired cellular migration, inadequate matrix remodeling, and, in some cases, microbial colonization or infection [4]. These challenges underscore the need for wound-care approaches that can reduce the microbial burden within wounds while promoting tissue repair.

Because wound repair is a multifactorial process, there is interest in developing therapeutic modalities that regulate more than one aspect of healing. In addition to covering the wound surface, a multifunctional wound-healing material could regulate inflammation, reduce oxidative stress, facilitate cell migration and proliferation, stimulate angiogenesis and extracellular matrix remodeling, and, when needed, reduce the microbial burden [5]. Nanomaterials are being investigated in regenerative medicine owing to their small size, high surface-area-to-volume ratio, tunable surface chemistry, and capacity to interact with biological interfaces. These properties may confer therapeutic benefits distinct from those of the same materials in their non-nanoscale forms [6]. Silver nanoparticles (AgNPs) are among the most studied nanomaterials for wound-related indications. Interest in AgNPs is driven primarily by their broad-spectrum antimicrobial and antibiofilm properties [7]. However, there is also growing evidence that AgNP formulations can influence inflammation, macrophage-associated immune responses, oxidative homeostasis, fibroblast and keratinocyte function, collagen deposition, and angiogenesis [8,9]. However, these biological effects depend on AgNP size, morphology, surface coating, silver ion release, dose, and duration of exposure [10].

The biological activity of AgNPs is strongly influenced by their physicochemical properties. The synthetic route is therefore important because it can affect both the physicochemical properties and the biological performance of AgNPs [11,12]. Although physical and chemical synthesis methods can produce AgNPs with well-controlled physicochemical properties, they often require substantial energy inputs, organic solvents, strong reducing agents, and stabilizing additives, raising concerns about costs, environmental impacts, and the toxicity of residual reagents. By contrast, the green synthesis of AgNPs offers an alternative that uses milder and more environmentally friendly conditions [12,13,14]. In this review, “plant-mediated synthesis” refers to AgNP production using plant-derived extracts, and the resulting nanoparticles are termed “plant-mediated AgNPs.” This approach exploits a variety of phytochemicals that can act as reducing, capping, or stabilizing agents [15]. Functional groups within plant-derived compounds, including phenolics such as flavonoids and tannins, as well as terpenoids, alkaloids, proteins, polysaccharides, sugars, and organic acids, can participate in the reduction of Ag^+^ ions, thereby promoting nanoparticle nucleation and growth while contributing to stabilization. Thus, plant extracts used in green synthesis can not only mediate nanoparticle formation but also influence nanoparticle size, shape, surface chemistry, colloidal stability, Ag^+^ ion release, and biological interactions [12,13,16].

Green-synthesized AgNPs are of interest for wound-healing applications because phytochemicals involved in the synthesis may also contribute to their biological performance. These phytochemicals may contribute antioxidant, anti-inflammatory, antimicrobial, or tissue-supportive activities [15,17]. For example, Pothireddy et al. reported that AgNPs synthesized using *Cinnamomum verum* extract yielded more favorable wound-healing outcomes than citrate-coated, chemically synthesized nanosilver and the plant extract alone in experimental wound models [18]. Ahmad et al. also observed that AgNPs synthesized using extracts from *Coriandrum sativum* exhibited stronger antibacterial, anti-inflammatory, antioxidant, and wound-healing effects than chemically synthesized AgNPs [19]. These studies suggest that differences in AgNP performance may reflect the combined effects of the AgNPs themselves and bioactive phytochemicals associated with the nanoparticle surface during plant-mediated synthesis. The extent of this contribution likely depends on plant type, plant part, extraction solvent, phytochemical profile, and synthesis parameters [15,18,19,20].

Alongside their therapeutic potential, the biosafety of AgNPs warrants careful consideration. Their toxicity is context-dependent and influenced by particle size and shape, surface chemistry and capping agents, aggregation state, dose and exposure duration, and the extent of Ag^+^ dissolution [21,22]. These factors can alter cellular interactions and silver bioavailability, potentially inducing cytotoxicity through excessive reactive oxygen species generation, oxidative stress, organelle dysfunction, inflammation, and apoptosis. Such effects may occur at the application site or, following systemic absorption and biodistribution, in distant tissues [21,22,23]. Although green synthesis may reduce the use of hazardous reagents and biogenic surface capping may improve biocompatibility, it does not inherently guarantee a safe final product [21]. This is exemplified by *Ehretia rigida* leaf-extract-mediated AgNPs, which promoted wound-relevant cellular responses at low concentrations but induced concentration-dependent cytotoxicity at higher concentrations [24]. Therefore, plant-mediated AgNPs intended for wound-healing applications and their formulations should undergo cytocompatibility testing in relevant skin cells, hemocompatibility assessment where blood contact is anticipated, and appropriate in vivo evaluation of local and systemic safety.

Despite the growing body of literature on plant-mediated AgNPs for wound healing, a clear scientific framework linking synthesis-related factors to biological performance remains insufficiently defined. Plant-mediated AgNPs are often evaluated as wound-healing agents without systematic investigation of many of the upstream variables that influence their physicochemical properties and biological performance. These variables include plant species, plant part, extraction solvent and method, phytochemical composition, AgNO_3_ concentration, extract-to-precursor ratio, pH, temperature, and reaction time. All of these factors may affect nanoparticle size, morphology, surface chemistry, colloidal stability, the Ag^+^ release profile, and ultimately cellular and tissue-level interactions and responses [25,26,27,28].

Another gap exists between nanoparticle synthesis and the development of clinically relevant formulations for AgNP delivery. AgNPs are most often tested as a standalone dispersion, whereas topical applications usually require incorporation into a formulation. The effects of formulation on AgNP stability, aggregation, release behavior, and wound-healing outcomes are rarely investigated comprehensively [29,30].

Moreover, studies tend to employ heterogeneous wound models and assess diverse endpoints using in vitro scratch assays, antimicrobial efficacy tests, and in vivo excision, incision, burn, diabetic, and infected wound models [31,32]. While the focus of many studies is wound closure or antimicrobial activity, the cellular and molecular basis of AgNP-mediated repair is not yet well understood [8]. Without standardization of botanical source, synthesis conditions, physicochemical characterization, formulation, dose, wound model, and mechanistic endpoints, it remains difficult to compare, interpret, or extrapolate study findings and to identify systems suitable for further translational evaluation.

**Figure 1:**
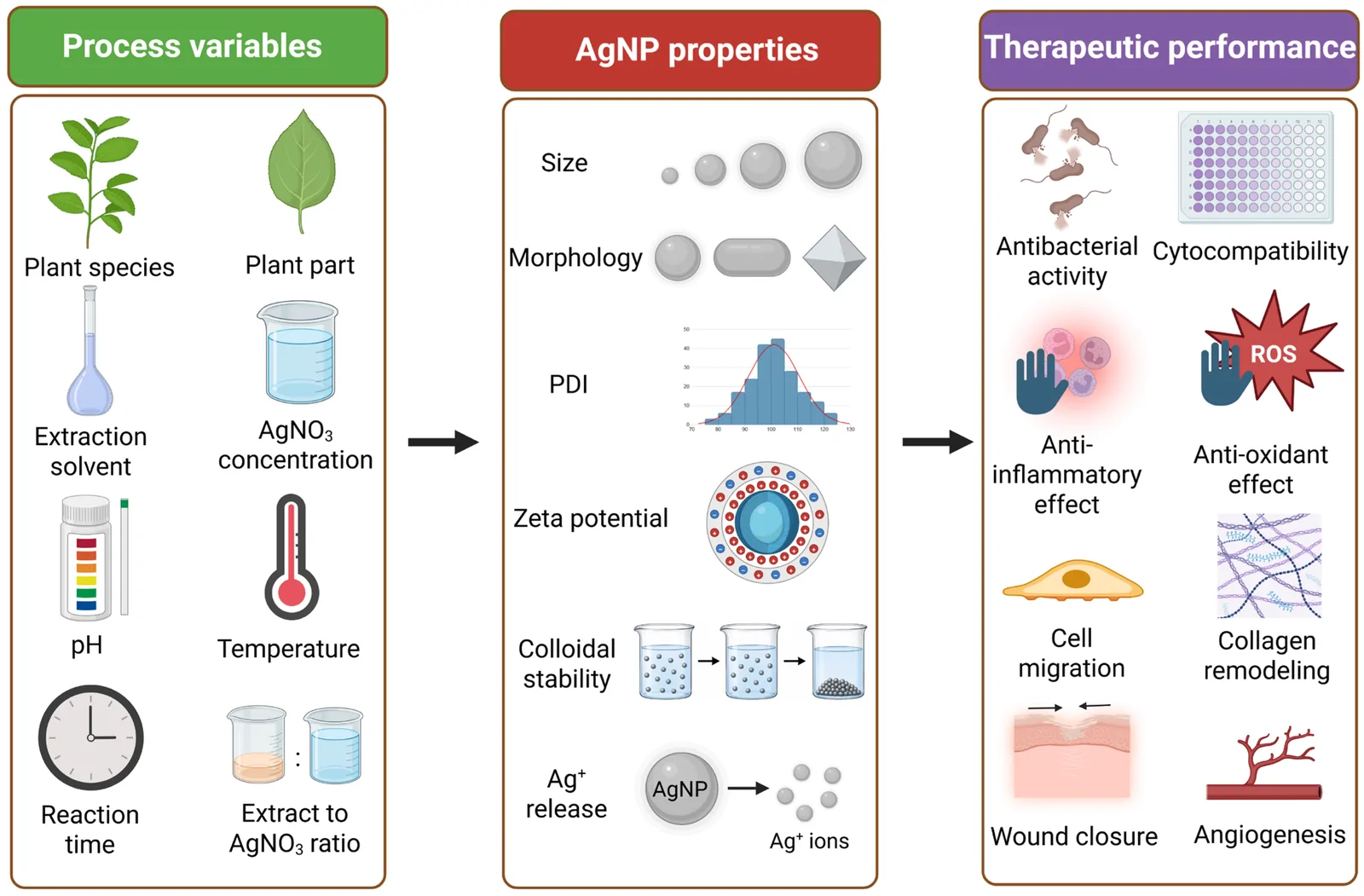
Process–property–performance framework for plant-mediated silver nanoparticles (AgNPs) intended for wound-healing applications. Process variables influence AgNP properties, which in turn shape therapeutic performance. Abbreviation: PDI, polydispersity index. Created in BioRender. Billa, N. (2026) https://BioRender.com/bwwgb1e, accessed on 24 August 2026.

Accordingly, this review critically evaluates recent evidence on plant-mediated AgNPs for wound-healing applications. It examines methodological trends in the selection of plant materials and extracts, synthesis approaches, and the optimization of reaction parameters, and considers how botanical source, extraction strategy, synthesis conditions, AgNP physicochemical properties, and formulation environment may influence wound-healing outcomes. These dimensions are integrated within a process–property–performance framework (Figure 1), together with a study-level assessment of which relationships among synthesis variables, AgNP properties, and therapeutic performance have been directly examined. The review also evaluates optimization endpoints, formulation strategies, administered AgNP exposure, cytocompatibility, experimental controls, active comparators, and in vivo study design, while discussing the reported antimicrobial, anti-inflammatory, antioxidant, pro-migratory, and pro-angiogenic effects and mechanisms associated with extracellular matrix remodeling. The main tables, together with Supplementary Tables S1–S3, consolidate detailed data on synthesis, characterization, formulation, exposure, safety, and biological outcomes. These data can serve as a practical starting point for designing future experiments, including selecting synthesis conditions, characterization endpoints, formulation strategies, comparator groups, dose-reporting methods, and safety assessments. Collectively, this approach identifies the methodological and translational gaps that currently limit reproducibility and comparison across studies.

PubMed and Scopus databases were used to conduct a targeted search for recent original articles focused on the plant-mediated synthesis of AgNPs for wound-healing applications. Keywords were mapped based on three concepts: plant-mediated synthesis, silver nanoparticles (AgNPs), and wound healing. Recent original research articles published since 2021 were reviewed. Selected publications were evaluated with particular attention given to synthesis-related parameters, the physicochemical properties of the synthesized AgNPs, formulation approaches, wound-healing studies, biological outcomes, and possible mechanisms. This approach was intended to support a critical synthesis of methodological trends and translational gaps rather than to provide a systematic review or meta-analysis.

## 2. Methodological Considerations and Trends

This section examines methodological parameters that may affect the plant-mediated synthesis of AgNPs. Each subsection first discusses the general impact of a factor on plant-mediated AgNP synthesis, followed by its specific implications for plant-derived AgNPs intended for wound healing. Trends in each variable among the studies included in this review are also highlighted.

### 2.1. Plant Species Selection

Several factors should be considered when selecting an appropriate plant source for the green synthesis of AgNPs. One relevant factor is the phytochemical profile of the plant. The plant species should contain primary and secondary metabolites capable of reducing Ag^+^ to elemental Ag, facilitating nanoparticle nucleation and growth, and capping the resulting AgNPs. These compounds include phenolics, flavonoids, tannins, terpenoids, alkaloids, proteins, polysaccharides, sugars, organic acids, and saponins, among others [12]. Consequently, plant extracts vary in their capacity to mediate AgNP synthesis. For example, Velgosová et al. compared several plant-derived extracts and showed that differences in extract composition affected the rate of Ag^+^ reduction, synthesis efficiency, nanoparticle properties, and stability [33]. They used the green microalga (*Parachlorella kessleri*) to synthesize AgNPs with sizes ranging from 5 to 60 nm [29]. Preliminary phytochemical indices can also help guide the selection of botanical sources for metal nanoparticle biosynthesis. For instance, total phenolic content (TPC) and total flavonoid content (TFC) are two indices that can be measured using relatively simple colorimetric assays. Higher TPC and TFC values indicate greater reducing and stabilizing potential, although they should not be interpreted as standalone predictors of AgNP quality. Rocha et al. used TPC and TFC as selection criteria to identify plant by-products capable of producing stable AgNPs [34].

A second important consideration is the reproducibility of the physicochemical properties of AgNPs synthesized from the selected plant material. Plants grown under standardized conditions are more likely to produce AgNPs with consistent properties than plants collected from different locations, where variations in water availability, soil composition, light exposure, temperature, and other environmental factors can markedly alter the phytochemical profile of the plant [35]. For example, *Paullinia cupana* extracts prepared from leaves collected during the dry and rainy seasons produced AgNPs with different surface plasmon resonance (SPR) peak intensities, hydrodynamic sizes, particle concentrations, size distributions, and biological activities. The authors attributed these differences to seasonal changes in the secondary metabolite composition of the extracts [36].

Another consideration is the long-term availability of the plant and the feasibility of its large-scale collection. For green synthesis to be commercially viable, it should be cost-effective, particularly for industrial-scale production [13]. If the plant source is scarce, not readily available, costly, or requires labor-intensive collection, it may be less suitable for mass production. The safety of plant-derived compounds is another important consideration. Some plant components may remain associated with AgNPs and exert their own biological effects. Therefore, plant sources with well-characterized safety profiles may be preferable.

The sustainability of the green extraction process should also be considered. Lower-impact extraction approaches include aqueous extraction, decoction, and infusion. If the extraction of plant constituents requires organic solvents, high temperatures, prolonged extraction times, or extensive purification, the extraction method may be less consistent with green-synthesis principles [37]. Finally, plants with documented biological activities that align with the intended biomedical application of the resulting AgNPs may be preferable. This is because bioactive compounds from the selected plant may remain associated with the nanoparticles as capping agents and exert their own biological effects. For instance, plant sources with documented antibacterial, antioxidant, or anti-inflammatory activities may be selected when these activities align with the intended application [13].

Across the studies reviewed here, a wide array of plants has been used to synthesize AgNPs intended for wound-healing applications, as summarized in Table 1. From a botanical perspective, all species except *Pyrrosia petiolosa*, a fern, were seed plants. Among the seed plants, *Pinus eldarica* was the only gymnosperm, whereas all the others were angiosperms. Within the angiosperm species, dicotyledonous plants constituted a larger proportion. When the plants were classified by family, Rosaceae was one of the most frequently represented plant families, represented by several fruit-bearing or medicinal species, including *Cydonia oblonga*, *Prunus avium*, *Aronia melanocarpa*, *Prunus spinosa*, and *Potentilla fulgens*. Plants within this family, especially edible ones, are well known to be rich sources of phenolics, flavonoids, tannins, anthocyanins, and organic acids [38]. This trend suggests a preference for polyphenol-rich botanical sources for AgNP synthesis for wound-healing applications. Another noticeable trend was the frequent use of medicinal and aromatic herbs, particularly those belonging to the families Lamiaceae, Asteraceae, and Apiaceae, which were represented in four, three, and three of the reviewed studies, respectively. Plants belonging to these families are typically rich in phenolics, flavonoids, terpenoids, and essential oil constituents [39].

When plant sources were classified by their functional use, several edible or food-derived plants were identified, including *Cydonia oblonga* (quince), *Punica granatum* (pomegranate), *Citrus aurantium* (bitter orange), *Actinidia deliciosa* (kiwifruit), *Camellia sinensis* (tea), *Olea europaea* (olive), *Coriandrum sativum* (coriander), and *Petroselinum crispum* (parsley), as shown in Table 1. This trend may reflect the accessibility, expected safety, and phytochemical richness of edible plant sources.

Another notable pattern across the reviewed articles is that many plants used to synthesize AgNPs for wound healing possess inherent antioxidant, antimicrobial, anti-inflammatory, or wound-healing-related activities. Representative examples include *Aloe vera*, *Curcuma longa* (turmeric), *Azadirachta indica* (neem), *Tridax procumbens* (coat buttons), *Punica granatum* (pomegranate), *Camellia sinensis* (tea), *Nigella sativa* (black seed), *Olea europaea* (olive), *Coriandrum sativum* (coriander), and *Petroselinum crispum* (parsley), as shown in Table 1 [40,41,42,43,44]. This pattern suggests that plant selection for wound-healing AgNP synthesis may be guided partly by the biological activities of the plants and that the phytochemicals involved in nanoparticle formation and stabilization may also contribute to the biological activity of the resulting product.

The majority of articles reviewed used a single plant extract to synthesize AgNPs. Only a few studies employed a combination of two plant extracts (Table 1). The use of systems incorporating multiple plant extracts may provide greater phytochemical diversity, with potential synergistic effects on Ag^+^ reduction, nanoparticle capping, stabilization, and wound-healing-related biological activity [45]. However, such systems may increase formulation complexity and reduce batch-to-batch reproducibility. They may also make it difficult to determine which plant-derived components contribute to nanoparticle formation and activity.

**Table 1:** Overview of plant-mediated AgNP synthesis parameters and physicochemical properties in the reviewed wound-healing studies. Abbreviations: AgNPs, silver nanoparticles; PDI, polydispersity index; NR, not reported; RT, room temperature.

Plant Species	Plant Family	Plant Part Used	Solvent for Plant Extraction	Synthesis Method	AgNO_3_ Stock Concentration	Extract to AgNO_3_ Ratio ^1^	pH	Temperature	Stirring Speed	Time	Size (nm) (Method)	PDI	Zeta Potential (mV)	Morphology	Study Year	Ref.
*Pyrrosia petiolosa* (Christ) Ching	Polypodiaceae	Leaves and roots	Distilled water	Conventional green synthesis	5886 mM ^2^	5000:1 *v*/*v*	NR	80 °C	200 rpm	2 h	10–120 (TEM)	NR	NR	NR	2026	[46]
*Pinus eldarica*	Pinaceae	Leaves	Deionized water	Conventional green synthesis	1 mM	NR	NR	NR (boiling condition)	NR	NR	NR	NR	NR	Irregular	2026	[47]
*Euphorbia hirta*; *Ocimum americanum*	Euphorbiaceae; Lamiaceae	Aerial parts	Deionized water	Microwave (800 W)-assisted green synthesis	1.03 mM	24.75:1 *v*/*v*	NR	70 °C	NR	12 min	102.5 ± 36 (SEM)	NR	−25	Spherical	2026	[48]
*Hibiscus rosa-sinensis*; *Cymbopogon citratus*	Malvaceae; Poaceae	Flowers; leaves	Distilled water	Conventional green synthesis	58.9 mM ^3^	1:33.33 *v*/*v*	NR ^4^	NR	NR	25 min	129.9 (DLS)	0.139	−28.6	Spherical	2026	[49]
*Cydonia oblonga* L.	Rosaceae	Leaves	Distilled water	Conventional green synthesis	5 mM	1:1 *v*/*v*	9	RT	NR	NR	60 ± 2 (SEM)	NR	NR	Spherical	2026	[50]
*Prunus avium* L.	Rosaceae	Stems	Methanol	Conventional green synthesis	15 mM	1:1 *v*/*v*	12	85 °C	NR	45 min	10–35 (TEM); 79.2 (DLS)	NR	+21.16	Spherical	2026	[51]
*Punica granatum*	Lythraceae	Fruit peels	Deionized water	Conventional green synthesis	2 mM	1:6.67 *v*/*v*	6.3	40 °C	NR	15 min ^5^	40–60 (FE-SEM)	NR	+72.1	Quasi-spherical	2026	[52]
*Aronia melanocarpa*	Rosaceae	Fruits	Ethanol 52%	In situ green synthesis hydrogel	20 mM	NR	NR ^6^	25 °C	NR	3 h	25.55 (DLS)	0.30 ± 0.05	−27.38 ± 2.38	Spherical	2025	[53]
*Tagetes erecta*	Asteraceae	Flowers	Distilled water	Conventional green synthesis	1 mM	1:9 *v*/*v*	NR	RT (~25 °C)	NR	24 h	311 (DLS)	0.5	−19.7	Clustered	2025	[54]
*Portulaca oleracea*	Portulacaceae	Leaves									304 (DLS)	0.5	−20.1	Spherical		
*Cyperus rotundus*	Cyperaceae	Roots	Double-distilled water	Conventional green synthesis	10 mM	1:5 *v*/*v*	NR	60 °C	500–600 rpm	1 h	633 (DLS); 288.52 (crystallite size, XRD)	0.483	−13.98	Spherical	2025	[55]
*Nigella sativa* L.	Ranunculaceae	Seeds	Chloroform	Conventional green synthesis	10 mM	1:9 *v*/*v*	NR	50 ± 1 °C	600 rpm	24 h	69.26 (DLS)	NR	NR	Spherical	2025	[56]
*Dendrobium officinale* Kimura et Migo	Orchidaceae	Stems	Water	Conventional green synthesis	60 mM	4:1 *v*/*v*	7	50 °C	NR	7 h	37.69 ± 10.13 (DLS)	NR	−13.3 ± 0.6	Spherical	2025	[57]
*Curcuma longa* L.	Zingiberaceae	Rhizomes	Ethanol	Plant extracts as reducing and sodium citrate as stabilizer	NR	1:10 *v*/*v*	NR	NR ^7^	NR	NR	10–40 (TEM); 39 ± 4 (DLS)	NR	−22 ± 3	Quasi-spherical	2025	[58]
*Euterpe oleracea*	Arecaceae	Fruits									10–40 (TEM); 34 ± 2 (DLS)	NR	−28 ± 3	Quasi-spherical		
*Lannea coromandelica*	Anacardiaceae	Bark	Deionized water	Conventional green synthesis followed by chitosan encapsulation	1 mM	1:9 *v*/*v*	NR	60 °C	NR	30 min	30–80 (TEM); 44.18 (crystallite size, XRD)	NR	−29.7	Spherical	2025	[59]
*Prunus spinosa*	Rosaceae	Fruits	Water	Green synthesis followed by hydrothermal treatment (120 °C, 12 h)	0.177 mM	1:1 *v*/*v*	NR	60 °C	600 rpm	1 h	~30 (TEM, and crystallite size by XRD)	NR	NR	Spherical	2025	[60]
*Citrus aurantium* L.	Rutaceae	Fruits	Double-distilled water	Conventional green synthesis	5 mM	1:9 *v*/*v*	10	90 °C	NR	1 h	5–32 (TEM); ~80 (DLS)	NR	−23.8	Spherical	2025	[61]
*Cassia sericea*	Fabaceae	Leaves	Distilled water	Conventional green synthesis	1 mM	1:9 *v*/*v*	NR	RT (20–22 °C)	NR	3 h	50.4 (DLS); 100–120 (SEM)	NR	NR	Tubular rod-shaped	2026	[62]
*Premna integrifolia* L.	Lamiaceae	Roots	Ethanol 60%	Sunlight-assisted green synthesis	1 mM	1:19 *v*/*v*	7	38 ± 2 °C	NR	10 min	34 (TEM)	NR	−11.5	Spherical	2024	[63]
*Colocasia esculenta*	Araceae	Corms	Distilled water for extraction	Sunlight-assisted green synthesis	5 mM	1:1 *v*/*v*	NR	NR	NR	15 min	244.9–272.2 (DLS)	0.530	−18.8	Spherical	2024	[64]
*Caralluma adscendens* R. Brown var. *bicolor*	Apocynaceae	Leaves	Double-distilled water	Microwave (40% power)-assisted green synthesis	100 mM	1:1 *v*/*v*	NR	NR	NR	3 min	17 (TEM)	NR	NR	Spherical	2024	[65]
*Aloe vera*	Asphodelaceae	Leaves	Sterile distilled water	Conventional green synthesis	10 mM	1:1 *v*/*v*	NR ^8^	NR	NR	24 h	28.82 (TEM)	NR	NR	Spherical to ellipsoidal	2024	[66]
*Camellia sinensis*	Theaceae	Leaves	Purified water	Conventional green synthesis	6 mM	1:9 *v*/*v*	NR	40 °C	NR	6 h	36.90 ± 1.22 (DLS); 20–50 (SEM)	0.049 ± 0.001	NR	Spherical to polygonal	2024	[67]
*Coriandrum sativum* L.	Apiaceae	Leaves	Double-distilled water	Conventional green synthesis	0.1 mM	1:10 *v*/*v*	NR	RT	NR	48 h	~167 (DLS); ~100 (TEM)	NR	−32.4	Cuboidal	2024	[19]
*Petroselinum crispum*	Apiaceae	Seeds	Methanol	Conventional green synthesis	15 mM	1:1 *v*/*v*	12	85 °C	NR	1.75 h	~150 (DLS); 30–50 (SEM/TEM); 38.7 (crystallite size, XRD)	NR	−23.2	Spherical	2023	[68]
*Camellia sinensis*; *Ocimum sanctum*	Theaceae; Lamiaceae	Leaves	Distilled water	Conventional green synthesis	1000 mM	1:1 *v*/*v*	10.5	NR	NR	NR	~7–7.5 (TEM)	NR	NR	Spherical	2023	[69]
*Actinidia deliciosa*	Actinidiaceae	Fruits	No extraction; fresh fruit juice	Sonication-assisted green synthesis; chitosan added as stabilizer	10 mM	1:1.29 *v*/*v*	NR	80 °C	NR	1.5 h ^9^	60.2 (DLS)	1.030	−22	Flaky nanocomposites	2023	[70]
*Potentilla fulgens*	Rosaceae	Roots	Ethanol 70%	Sonication-assisted green synthesis	3 mM	1:9 *v*/*v*	NR	35 °C	200 rpm	1.25 h ^10^	15–20 (TEM); 137 (DLS)	0.206	−25.5	Spherical	2023	[71]
*Camellia sinensis*; *Olea europaea* L.	Theaceae; Oleaceae	Leaves	Distilled water	Conventional green synthesis	10 mM	NR	8	RT	NR	20 min	6.87–8.51 (TEM); 88 ± 1.6 (DLS)	0.179 ± 0.02	NR	Spherical	2022	[72]
*Rhizophora apiculata*	Rhizophoraceae	Leaves	Distilled water	Conventional green synthesis	1 mM	1:10 *v*/*v*	NR	RT (26–27 °C)	NR	4 h	35–100 (SEM); 99.6 (DLS)	1.752	−6	Irregular	2022	[73]
*Scutellaria barbata*	Lamiaceae	NR	Deionized water	Conventional green synthesis	1 mM	1:9 *v*/*v*	NR	NR	NR	30 min	20–40 (TEM); 30–40 (AFM)	NR	NR	Spherical	2021	[74]
*Gnaphalium polycaulon*	Asteraceae	Leaves	Methanol	Conventional green synthesis	1 mM	1:9 *v*/*v*	NR	RT	NR	1 h	<50 (SEM/TEM); 25 crystallite size, XRD)	NR	NR	Spherical and triangular	2021	[75]
*Echinophora platyloba* DC	Apiaceae	NR	Double-distilled water	Conventional green synthesis; followed by Chloroxine conjugation	100 mM	3:1 *v*/*v*	NR	75 °C	NR	2 h	19.77 ± 1.06 (TEM); 19.50 (DLS); 30.19 (crystallite size, XRD) ^11^	0.353 ^12^	−21.6 ^13^	Spherical ^14^	2021	[76]
*Tridax procumbens* L.	Asteraceae	Leaves	Ultrapure type 1 water	Conventional green synthesis	3 mM	1:9 *v*/*v*	NR	80 °C	50 rpm	NR	138.0 ± 2.1 (DLS); 65–100 (SEM)	0.460 ± 0.3	−20.4 ± 5.20	Spherical	2021	[77]
*Azadirachta indica*	Meliaceae	Leaves	Distilled water	Conventional green synthesis	1 mM	1:9 *v*/*v*	7	RT (25 °C)	200 rpm	18 h	33.20 ± 3.79 (SEM/TEM)	NR	NR	Spherical	2021	[78]

^1^ Extract:AgNO_3_ ratios were retained when one side of the ratio was already 1. When neither side was 1, the smaller value was normalized to 1 while preserving the extract:AgNO_3_ order. ^2^ 1 g/mL. ^3^ 0.01 g/mL. ^4^ pH adjusted using NaOH; exact pH NR. ^5^ Then 10 days in dark in steady-state for complete reaction. ^6^ Alkaline; exact pH NR. ^7^ Heated to 90 °C, then cooled during reaction (inconstant temperature during synthesis). ^8^ Alkaline; exact pH NR. ^9^ 30 min of sonication followed by 1 h of heating. ^10^ 15 min sonication followed by 1 h dark incubation. ^11^ Chloroxine-conjugated AgNPs: 68.81 ± 3.63 (TEM), 70.80 (DLS), 20.35 (crystallite size, XRD). ^12^ Chloroxine-conjugated AgNPs: 0.973. ^13^ Chloroxine-conjugated AgNPs: −47.1. ^14^ Chloroxine-conjugated AgNPs: flower-like.

### 2.2. Plant Part Selection

In addition to the plant species, the plant part is important for the sustainable green synthesis of nanoparticles because different plant parts contain varying types and quantities of phytochemicals. Therefore, they vary in their capacity for Ag^+^ reduction and AgNP capping. In addition to nanoparticle yield, the use of different plant parts can influence the physicochemical properties and biological activity of the resulting AgNPs. For instance, *Macrotyloma uniflorum* leaf, root, stem, and seed extracts yielded AgNPs with distinct physicochemical properties [79]. According to Lima et al., AgNPs prepared using extracts from different parts of *Paullinia cupana* exhibited plant-part-dependent physicochemical and antibacterial properties. The authors profiled the phytochemical constituents of various organs using ultra-high-performance liquid chromatography (UHPLC) and high-resolution tandem mass spectrometry (HRMS) and attributed the differences observed among the resulting AgNPs to organ-specific phytochemical variation [80].

Among the reviewed articles, leaves were the most commonly used plant material for extract preparation, followed by fruit-derived materials such as whole fruits and fruit peels (Table 1). The predominance of leaf use may be related to the ready availability, renewability, relatively high biomass, and rich phytochemical content of leaves [81]. The frequent use of fruit-derived materials may be related to growing interest in edible, nutraceutical, and plant-waste resources as green synthesis platforms for AgNP production [82]. Extracts from roots, bark, rhizomes, corms, stems, seeds, and flowers were used less frequently, possibly because some of these organs are less abundant, less renewable, or more difficult to collect sustainably.

### 2.3. Extraction Solvent

The extract’s composition depends on the solubility threshold of phytochemicals in the solvent used for extraction. Specifically, solvent polarity can influence the type and quantity of phytochemicals extracted, as compounds with different polarities may dissolve selectively in different solvent systems [83]. Consequently, variation may occur in the efficiency with which the extract synthesizes AgNPs, as well as in the resulting nanoparticles’ properties. For instance, *Duchesnea indica* extracts prepared in water, ethanol, and dimethyl sulfoxide (DMSO) formed AgNPs with variable antibacterial activity profiles [84]. Additionally, AgNPs synthesized using aqueous, ethanolic, and diethyl ether extracts of *Waltheria americana* roots displayed different SPR positions and antimicrobial activities [85]. Similarly, AgNPs synthesized using aqueous, ethanolic, and methanolic root extracts of *Morus alba* displayed different biological activities [86]. Although no general aqueous-versus-organic trend can be established, some reports suggest that extracts prepared using organic or semi-organic solvents may produce smaller, less aggregated, or more biologically active AgNPs under specific experimental conditions [84,85,87].

The majority of the studies reviewed here employed aqueous extraction, as summarized in Table 1, which is consistent with the principles of green synthesis, because water is inexpensive, safe, biocompatible, and environmentally friendly [88]. However, the water grades used ranged from distilled and deionized water to ultrapure water. Such variation may influence the chemical composition of the extract and the reproducibility of AgNP synthesis. For example, the ionic composition of water, especially chloride ions, can affect the morphology of AgNPs [89]. Organic solvents were used less frequently and primarily included ethanol or hydroethanolic solutions. Methanol and chloroform were rarely used and were generally considered less compatible with green synthesis principles than aqueous extraction [90]. Nevertheless, the use of ethanol or hydroethanolic mixtures may broaden the phytochemical profile of the extract by improving the recovery of less polar compounds. In contrast to conventional extraction, only one study employed solvent-free juice derived from kiwi fruit for plant-mediated AgNP synthesis [70].

### 2.4. Green Synthesis Approaches

In this review, the term “green synthesis” refers to the procedural and operational steps used for AgNP fabrication using plant extracts. As illustrated in Table 1, among the reviewed articles, the most frequent method was conventional green synthesis, which involves mixing a silver nitrate precursor solution with a plant extract and incubating the mixture at room temperature or heating it, with or without stirring. In such an approach, phytochemicals in the plant extract may act as both reducing and capping agents [81]. This strategy, which may be considered a one-pot approach in many cases, avoids the use of external energy inputs other than the thermal energy provided by simple heating. This approach is well known for its relative simplicity, low cost, and accessibility [12].

By contrast, microwave-, sunlight-, and sonication-assisted methods were reported less frequently (Table 1). These methods typically require shorter reaction times and require energy input during AgNP synthesis [15,91]. Beyond shortening the reaction time, such assisted methods may also influence the physicochemical characteristics of the synthesized AgNPs. For instance, AgNPs synthesized using *Trigonella hamosa* leaf extract were slightly smaller when prepared under microwave-assisted conditions than under conventional synthesis conditions [92]. Moreover, AgNPs synthesized using *Lantana camara* flower extract varied in particle size, morphology, SPR features, and biological activity when prepared using reflux, microwave-assisted, or ultrasound-assisted treatments [93]. However, given the heterogeneity in plant source and reaction parameters, no conclusive comparison among these approaches can be made. Head-to-head comparisons under controlled reaction conditions should be performed to establish the impact of additional energy inputs on the rate, efficiency, and yield of green AgNP synthesis, as well as on the resulting nanoparticles’ properties.

## 3. Optimization of Reaction Parameters

As in the chemical synthesis of AgNPs, the fundamental chemical mechanism of green synthesis involves a redox process whereby Ag^+^ is reduced to Ag^0^ by the plant components, followed by nucleation, particle growth, capping, and stabilization. Therefore, parameters that can potentially affect the redox reaction may also affect the green synthesis process. Parameters that affect crystal growth and particle size are also important. These include pH, temperature, AgNO_3_ concentration, the ratio of AgNO_3_ to the plant extract, reaction time, and stirring speed during the reaction. Several studies on plant-mediated AgNP synthesis have optimized these parameters to improve AgNP formation, colloidal stability, or size control [26,94,95].

These optimization efforts can generally be categorized into two distinct strategies: one-factor-at-a-time (OFAT) and response surface methodology (RSM). In OFAT, optimization is conducted through several sequential steps. In each step, only one variable is changed, while the others are kept constant. After an optimal level is selected for one factor, the subsequent set of experiments is conducted to optimize the next variable. In contrast, RSM is a statistical method used to optimize experimental conditions by evaluating the effects of multiple variables simultaneously and identifying their interactions. OFAT is simple and easy to implement; however, it is time-consuming and cannot reveal interactions between variables. RSM, by contrast, evaluates multiple factors simultaneously and can identify both individual and interaction effects, making it more efficient and statistically robust for optimization [96]. Across the reviewed articles on plant-mediated synthesis of AgNPs intended for wound healing, six used OFAT optimization and one used RSM, as summarized in Table 2. Additionally, the synthesis parameters for all reviewed studies of plant-mediated AgNPs intended for wound healing were extracted and summarized in Table 1. The subsequent subsections primarily examine the influence of each synthesis parameter in the studies of plant-mediated AgNPs for wound healing included in this review. Selected examples from the broader plant-mediated AgNP literature are also included to discuss potential mechanisms and identify similar or contrasting trends.

Optimization of synthesis conditions was performed primarily using UV–Vis spectroscopy and SPR spectral features. Higher, sharper, and more characteristic SPR peaks were often taken as evidence of a more efficient AgNP synthesis process. Size, polydispersity index (PDI), zeta potential, transmission electron microscopy (TEM), scanning electron microscopy (SEM), and X-ray diffraction (XRD) analyses were typically used after optimization to characterize the chosen AgNPs, rather than being used as primary endpoints during optimization. An exception to this trend was the study by Karthikeyan et al., in which a Box–Behnken design was applied with particle size as the formal response variable [48]. Overall, synthesis optimization within the reviewed wound-healing AgNP literature was typically spectroscopy-driven, whereas systematic size-driven or bioactivity-driven optimization was uncommon.

### 3.1. Effect of AgNO_3_ Concentration

AgNO_3_ concentration generally exhibited an optimum-dependent effect across the reviewed studies, whereby modest increases at lower concentrations enhanced nanoparticle formation and increased SPR intensity up to an optimal level. Beyond that point, SPR intensity decreased or the peak quality deteriorated, as reflected by peak broadening and particle aggregation. In other words, insufficient AgNO_3_ did not promote nanoparticle formation, whereas excessive AgNO_3_ caused SPR peak displacement, often accompanied by peak broadening, a broader particle size distribution, or particle aggregation [81,94,97]. These observations may be explained by uncontrolled nucleation and growth or an insufficient number of capping agents relative to the available Ag^+^ at higher precursor concentrations. Consistent with these observations, other studies have reported that AgNO_3_ concentration can affect the size distribution and colloidal characteristics of plant-mediated AgNPs [98,99].

**Figure 2:**
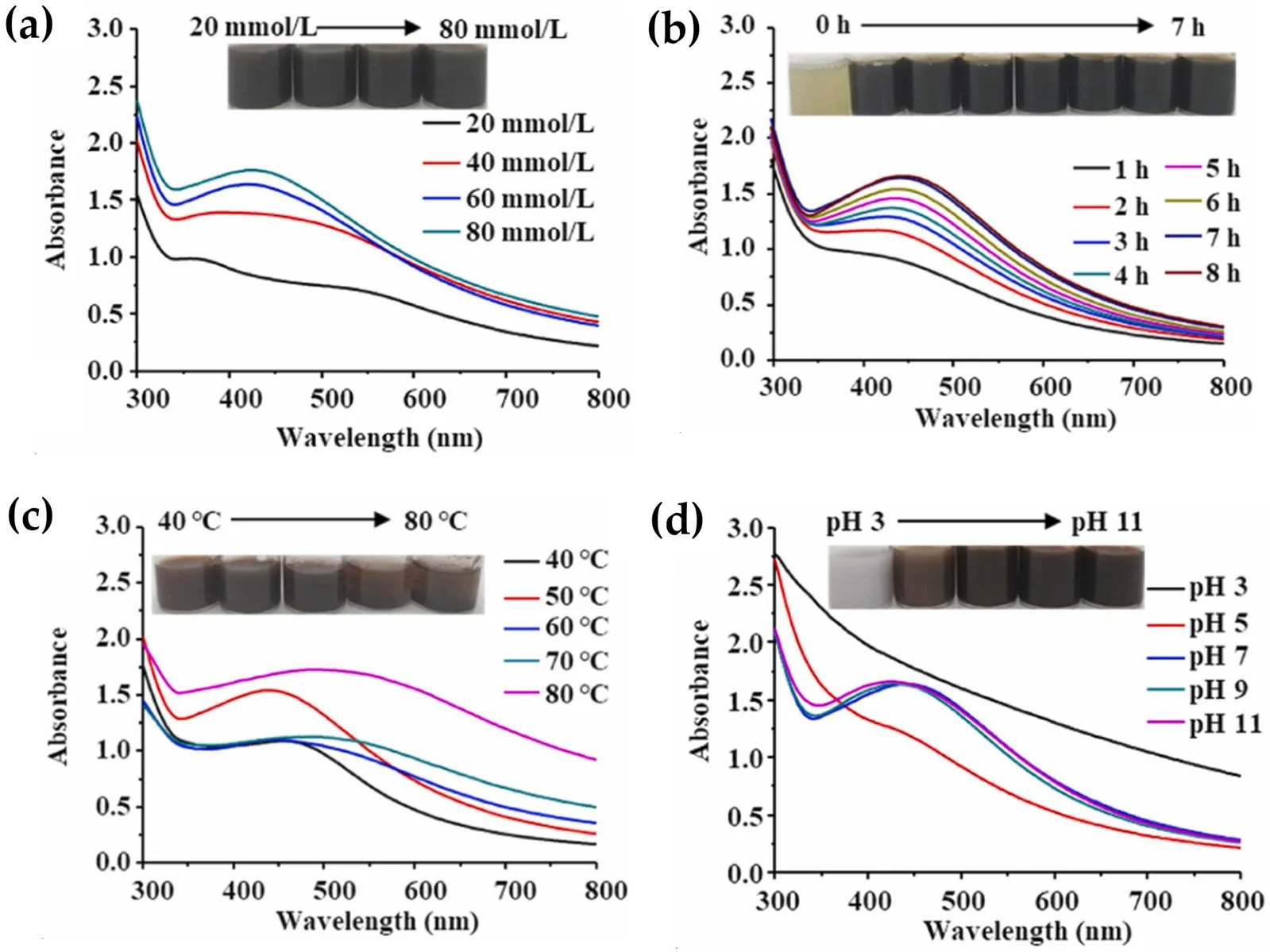
UV–Vis absorption spectra illustrating the effects of (**a**) AgNO_3_ concentration, (**b**) reaction time, (**c**) reaction temperature, and (**d**) pH on the green synthesis of *Dendrobium officinale* extract-mediated silver nanoparticles (AgNPs). Visual observations of the as-prepared samples are shown as insets in their corresponding panels. Panels were cropped, rearranged, and relabeled from Lv et al. [57] under the terms of the Creative Commons Attribution 4.0 International License (CC BY 4.0).

In the wound-healing studies reviewed here, the optimal AgNO_3_ concentration varied depending on the plant source. For example, Zare-Bidaki et al. and Dehghan et al. reported 15 mM AgNO_3_ as the optimal precursor concentration, whereas Ahmad et al. reported 6 mM as the optimal concentration [51,67,68]. Although Lv et al. reported the sharpest SPR peak at 80 mM, 60 mM was selected as the optimal concentration because of nanoparticle precipitation at 80 mM (Figure 2a) [57]. In the RSM-based optimization conducted by Karthikeyan et al., an increase in AgNO_3_ precursor concentration was associated with larger AgNP particle sizes, and precursor concentration affected particle size to a greater extent than an increase in the plant extract ratio [48]. Hence, AgNO_3_ concentration should be carefully tuned for each plant species because slight variations in Ag^+^ concentration may hinder AgNP formation and affect particle size.

### 3.2. Effect of Reaction Time

Reaction time also showed a time-dependent pattern with an optimum, whereby longer reaction times generally caused an increase in the yield of AgNPs, as evidenced by a higher SPR signal. Reaction times beyond the optimal time did not improve the quality of the AgNPs and were associated with increased particle size or a loss of SPR intensity. For example, AgNPs synthesized using *Garcinia mangostana* fruit extract exhibited the highest UV–Vis absorbance at an intermediate reaction time, whereas extended incubation periods exceeding 5 h reduced absorbance and were associated with particle aggregation [100]. In *Azadirachta indica* leaf extract-mediated AgNP synthesis, absorbance increased up to an optimal reaction time and subsequently decreased with prolonged incubation [26]. Ahmad et al. observed no distinct SPR peak for *Camellia sinensis*-mediated AgNPs after 1 h, a small peak after 2 h, and apparent completion of the reaction by 6 h. However, extending the reaction time to 24 h resulted in an increase in particle size [67]. Zare-Bidaki et al. also identified 105 min as the optimal reaction time for *Petroselinum crispum*-mediated AgNP synthesis, and reported decreased absorbance with longer reaction times [68].

Dehghan et al. found that increasing the reaction time from 15 to 45 min increased the SPR intensity of *Prunus avium*-mediated AgNPs, whereas no marked change was observed at 60 min [51]. Similarly, in the study by Lv et al., *Dendrobium officinale*-mediated synthesis of AgNPs showed a progressive increase in absorbance up to 7 h, with no further significant increase at 8 h (Figure 2b) [57]. Overall, sufficient reaction time is required for Ag^+^ reduction, but excessive incubation can cause a loss of particle colloidal stability or control over particle size.

### 3.3. Effect of Reaction Temperature

Increasing reaction temperature usually accelerates AgNP synthesis and promotes the development of more intense SPR peaks [101]. This effect is likely related to faster reduction kinetics, as well as increased nucleation and growth rates. However, temperature should be regarded as an optimum-dependent parameter rather than one with a continuously favorable effect. Although heating can increase the rate of Ag^+^ reduction and AgNP formation, excessive heating may drive nucleation and growth beyond the stabilizing capacity of plant-derived capping agents, resulting in broad SPR bands, larger particles, aggregation, or poorer colloidal quality. For instance, AgNPs synthesized by Afshin et al. using a polyherbal formulation exhibited the sharpest SPR peak at 25 °C. Increasing the temperature to 100 °C resulted in peak broadening, suggesting poorer nanoparticle quality at higher temperatures in that system [102].

Another possible reason for this optimum-dependent effect of temperature is the potential degradation of active compounds in the plant extract above a certain temperature. For instance, Zhang et al. showed that increasing the reaction temperature to 90 °C enhanced the UV–Vis absorption peak and promoted *Dianthus superbus*-mediated AgNP synthesis. However, further increases in temperature reduced the absorption peak, which the authors attributed to possible heat-induced damage to bioactive compounds, thereby impairing AgNP reduction and stabilization [103]. Likewise, during the synthesis of AgNPs with *Artemisia argyi* extract, a temperature of 60 °C allowed the formation of the most symmetric SPR peak. Temperatures above this optimum were considered unfavorable because of the potential decomposition of extract biomolecules and nanoparticle instability [104]. However, this association was based on the general concept of the heat susceptibility of some phytochemicals and needs to be confirmed using appropriate assays.

Conversely, increasing temperature does not always correlate with aggregation or decreased colloidal quality. For example, during *Mitragyna parvifolia*-mediated AgNP synthesis, although the SPR intensity at 90 °C was lower than that observed at 30 °C, a lower-wavelength SPR band and a smaller hydrodynamic particle size were observed at the higher temperature. This suggests that a higher temperature may favor the formation of smaller AgNPs rather than aggregation in some extract systems. This could be attributed to temperature-induced acceleration of Ag^+^ reduction and nucleation. Faster reduction kinetics may favor the rapid formation of smaller nuclei before particle growth and aggregation occur to a substantial extent [105].

Temperature was optimized in some of the studies included in this review. For instance, Zare-Bidaki et al. and Dehghan et al. found that increasing the reaction temperature from room temperature to 85 °C led to increased SPR intensity, and 85 °C was selected as the optimal temperature [51,68]. Chang et al. also noted that higher temperatures produced more intense and narrower SPR peaks and chose 90 °C as the optimal temperature [61]. However, Ahmad et al. selected 40 °C instead of 100 °C, despite the reaction mixture turning gray much faster at 100 °C, indicating more rapid completion of the reaction. This choice was dictated by the larger hydrodynamic particle size and poorer PDI observed at 100 °C [67]. In the study by Lv et al., increasing the temperature from 40 °C to 50 °C resulted in increased SPR intensity. However, temperatures above that level caused peak broadening and a red shift, as shown in Figure 2c. This observation was attributed to reduced colloidal stability and increased aggregation at higher temperatures [57]. In general, elevated temperatures often increase the rate of AgNP formation. However, the effects of temperature are primarily reflected in the quality of the SPR profile, whereas particle size distribution, aggregation, and colloidal stability depend greatly on the plant extract system, its phytochemical thermal stability, and other concurrent reaction parameters.

### 3.4. Effect of pH

pH was another parameter optimized in only two of the studies reviewed here. Generally, alkaline conditions appeared to enhance AgNP formation. This effect may be attributable to the deprotonation of phenolic, flavonoid, and hydroxyl-containing phytochemicals, which may enhance their electron donation and reducing ability [106]. However, excessive alkalinity may decrease SPR intensity or promote aggregation. For instance, in the study by Chang et al., AgNP formation was promoted under alkaline conditions; therefore, pH 10 was selected. When pH was set above 10, the absorbance decreased drastically, suggesting nanoparticle aggregation [61]. With regard to pH selection for the green synthesis of AgNPs, SPR peak intensity should not be the only factor considered. Particle size and the compatibility of the selected pH with the final biomedical application must also be taken into account. For example, Lv et al. showed no absorbance at the acidic pH values of 3 and 5, whereas pH 7, 9, and 11 produced intense SPR peaks. Increasing pH from 7 to 11 caused a blue shift, suggesting smaller particle sizes (Figure 2d). Nevertheless, pH 7 was selected, partly because neutral pH was considered better suited for wound-healing applications [57]. The apparently contrasting observations reported by Lv et al. and Chang et al., namely a blue shift in one study and aggregation at higher pH in the other, illustrate the heterogeneous effect of pH on plant-mediated AgNPs and highlight the need to optimize pH for each plant species and extract system used in green synthesis.

Similar pH-dependent trends have also recently been documented in plant-mediated AgNP synthesis experiments. *Terminalia ferdinandiana* leaf extract failed to form AgNPs under strongly acidic conditions. At pH 8, nanoparticle formation occurred, and the resulting AgNPs exhibited decreased size and PDI [107]. Similarly, SPR spectra obtained during *Pergularia tomentosa*-mediated AgNP synthesis showed no peak between pH 4 and 6. Increasing pH to 9 resulted in a significant increase in SPR intensity and was accompanied by a blue shift in the SPR peak, implying better AgNP formation at alkaline pH values [108]. A similar pH-dependent pattern with an optimum was noted with *Piper chaba*-mediated AgNPs, with SPR intensity increasing as pH increased to 8 but decreasing with further increases in pH, possibly because of nanoparticle aggregation [109].

Universal conclusions about the role of pH in plant-mediated AgNP synthesis cannot be drawn, as pH appears to be highly system-dependent. On the one hand, mildly to moderately alkaline reaction environments generally favor Ag^+^ reduction and SPR development. On the other hand, the choice of pH should be based not solely on SPR intensity but also on particle size, PDI, the tendencies toward aggregation and agglomerate formation, colloidal stability, green chemistry considerations, and compatibility with the targeted biomedical application.

### 3.5. Effect of Extract Amount or Extract-to-AgNO_3_ Ratio

The extract-to-AgNO_3_ ratio exhibits another optimum-dependent pattern. Plant extracts introduce both reducing and capping phytochemicals into the reaction mixture; therefore, insufficient extract amounts may limit the ability of these phytochemicals to reduce Ag^+^ ions or stabilize the resulting nanoparticles. Conversely, an excessive amount of extract introduces more phytochemicals than necessary, resulting in the broadening of the SPR peaks. Additionally, excess plant-derived material may sterically hinder nucleation and growth processes, potentially leading to nanoparticle aggregation. This pattern was reported by Lv et al., who found that increasing the extract concentration from 5 to 20 mg/mL increased the SPR response. However, an extract concentration of 20 mg/mL was also associated with instability and precipitation. Therefore, 15 mg/mL was selected as the optimal extract concentration [57]. Ahmad et al. tested extract-to-AgNO_3_ ratios of 1:9, 2:8, and 5:5 and selected the 1:9 ratio because it produced the highest SPR absorbance [67]. Chang et al. evaluated extract amounts ranging from 0.2 to 1.0 mL and observed the highest SPR peak with 0.6 mL of extract, which corresponded to an extract-to-AgNO_3_ ratio of 1:9 [61]. In an RSM study, Karthikeyan et al. showed that the plant extract ratio interacted with other synthesis factors rather than acting independently [48].

Optimum-dependent behavior of the extract-to-AgNO_3_ ratio has also been documented in other recent studies. Increasing the extract-to-AgNO_3_ ratio in *Pergularia tomentosa*-mediated AgNP synthesis increased SPR intensity up to a threshold [108]. Similarly, *Piper chaba*-mediated AgNP synthesis showed a continuous increase in SPR intensity with increasing extract volume up to 2 mL. Above this threshold, a decrease in peak intensity was observed, followed by a slight red shift caused by aggregation [109]. In *Senna italica*-mediated synthesis, an increase in leaf extract volume beyond 2 mL yielded no further improvement [110]. Overall, these observations suggest that there is an optimal balance between extract concentration and Ag^+^ ions. Insufficient extract may limit the reducing and stabilizing potential of the reaction mixture, whereas excessive extract may adversely affect nucleation and growth.

## 4. Wound-Healing Applications of Plant-Mediated AgNPs

### 4.1. Formulation Strategies for Wound-Healing AgNPs

Although plant-mediated AgNPs are inherently bioactive, their direct application to wounds may be limited by limited residence time on the wound bed and inconsistent contact with damaged tissue. Controlling the release of Ag^+^ may also be difficult without an appropriate delivery system, and substantial direct exposure of wound tissue to nanoparticles may increase the risk of cytotoxicity [111]. AgNPs used without an added delivery material may also be more prone to aggregation. Therefore, formulation is important for translating plant-mediated AgNPs from bioactive agents into dosage forms suitable for wound application. Suitable formulations can improve wound retention, maintain a moist wound environment, improve spreadability and wound coverage, and provide an antimicrobial barrier [112,113,114]. They can also influence nanoparticle and silver-ion release. Polymeric or semisolid delivery systems may further support wound repair by absorbing exudate, facilitating cell migration, and providing occlusive and hydrating effects.

To classify the systems consistently, we used the physical form tested in each publication rather than the terminology used by the original authors. Each publication was counted once according to the main AgNP system evaluated for wound healing. Topical gels and hydrogels were combined as gel-based formulations because the studies did not provide consistent physical or rheological criteria for separating them. Dressings/bandages included self-supporting materials such as films, nanofibers, and multilayer constructs, whereas ointments were systems prepared in an ointment base. Chitosan-encapsulated AgNPs and polymer–AgNP nanocomposites that were not presented as a gel, dressing, or ointment were grouped as polymer-associated AgNP systems without a defined dosage form. AgNPs tested directly without an added delivery material were placed in a separate category.

**Figure 3:**
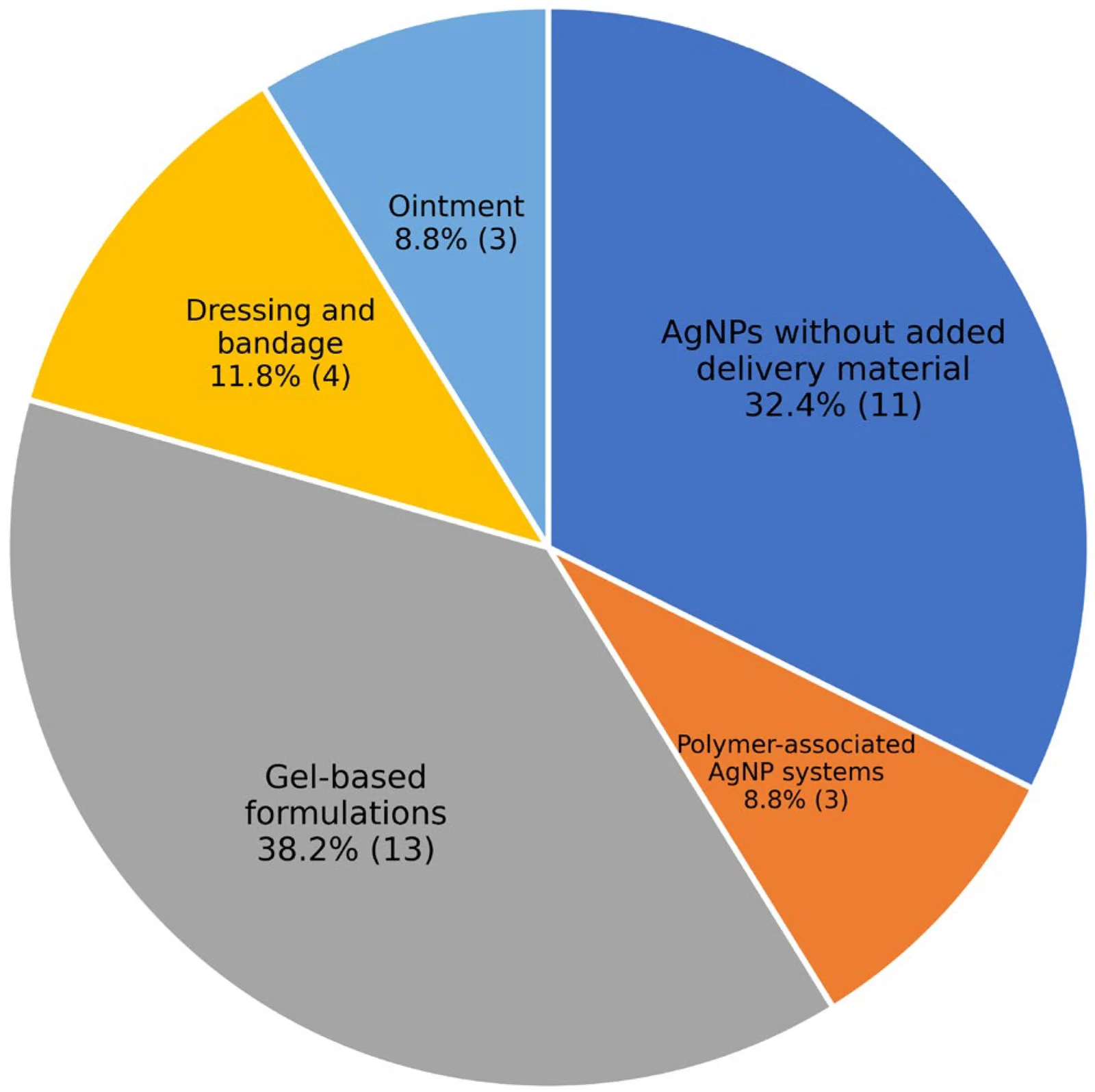
Distribution of plant-mediated AgNP systems according to their wound-delivery format among the 34 included publications. Each publication was counted once and assigned using the operational criteria described in Section 4.1.

Using this classification, gel-based formulations were reported in 13 publications (38.2%), followed by AgNPs without an added delivery material in 11 (32.4%), dressings/bandages in four (11.8%), polymer-associated AgNP systems without a defined dosage form in three (8.8%), and ointments in three (8.8%) (Figure 3). In the discussion below and in Table 3, the terms “hydrogel” and “topical gel” are retained only when referring to how individual systems were described in the original publications. The formulations and doses of plant-mediated AgNPs used for wound healing are summarized in Table 3.

AgNPs without an added delivery material were commonly used in early-stage antibacterial studies, scratch assays, and in vivo proof-of-concept experiments. Testing AgNPs directly avoids the influence of a separate delivery vehicle and can simplify the initial assessment of the complete nanoparticle system. However, it does not by itself establish the specific contribution of the phytochemical corona, which requires appropriate extract-only and chemically synthesized AgNP controls, as discussed in Section 6.5. Directly administered AgNPs also have limitations for wound delivery, including poor retention, aggregation, and limited control over local exposure and Ag^+^ release [7].

Within the combined gel-based category, several systems were described by the original authors as hydrogels. These systems were prepared using polymers such as Carbopol, hyaluronic acid (HA), citrus pectin, carboxymethylcellulose (CMC), and Poloxamer (Table 3). Hydrogels are attractive platforms for wound-healing applications because they offer several desirable properties, including high water content, moisture retention, wound conformability, swelling capacity, and the potential for controlled or stimuli-responsive release of therapeutic agents [115]. In some studies, hydrogels provided additional desirable properties such as self-healing behavior, adhesive properties, or enhanced exudate management [53,61,72].

The stimuli-responsive behavior of hydrogels may support wound healing by enhancing local retention and modulating the release kinetics of AgNPs or Ag^+^ at the wound site. The pH- and hyaluronidase-responsive HA hydrogel developed by Wang et al. exhibited accelerated AgNP release under acidic conditions and underwent hyaluronidase (HAase)-triggered degradation, thereby enhancing activity against methicillin-resistant *Staphylococcus aureus* (MRSA), promoting tissue remodeling, and reducing inflammation [53]. Thermosensitive Pluronic F127 hydrogels generally remain injectable or spreadable at lower temperatures but undergo gelation at skin temperature, thereby enhancing wound residence time and prolonging silver-ion release [63,78]. Although hydrogels offer several useful properties, challenges associated with their practical use include sterilization, storage stability, reproducibility, and scale-up [116].

Other systems within the same gel-based category were described by the original authors as topical gels and were prepared using polymers such as chitosan and Carbopol (Table 3). Their advantages include ease of application, better contact with the wound bed, higher local retention, and potentially more homogeneous AgNP delivery [117]. AgNP-loaded chitosan-based gels may additionally provide hemostatic, antimicrobial, mucoadhesive, and wound-supportive properties [118]. In the infected wound model used by Ahmad et al., for example, the nanoparticle-free chitosan gel significantly promoted tissue regeneration and inhibited inflammatory infiltration compared with untreated wounds. However, incorporating AgNPs into the gel resulted in greater tissue regeneration [67]. Despite the advantages of topical gel formulations, they may suffer from limited mechanical stability, and possible dilution or displacement in highly exudative wounds [119].

Dressings and bandages, including cotton fabrics, films, patches, electrospun nanofibers, and other wound-contact platforms (Table 3), generally provide stronger physical barriers than gels and hydrogels. These systems can thereby help prevent microbial entry. In addition to maintaining prolonged contact with the wound surface, AgNP-loaded dressings may reduce the frequency of dressing changes. Electrospun nanofibers and film-like matrices may also provide structures that resemble extracellular matrix (ECM) architecture and offer a high surface area for AgNP loading [119,120]. However, dressings and bandages may be less conformable to irregularly shaped wounds. Sterilization requirements, storage stability, reproducibility, and scale-up are also practical constraints that must be addressed before clinical translation [121].

Ointment-based AgNP preparations were less common among the reviewed studies (Table 3). Ointments are commonly used in clinical wound-care settings and are relatively simple to formulate. In the reviewed studies, the ointment bases were predominantly oleaginous: two studies used Vaseline-based ointments, whereas one used a combined base of Calendula flower oil and Vaseline oil [51,68,76]. Importantly, oleaginous bases such as Vaseline may occlude the wound, reduce transepidermal water loss (TEWL), and protect the wound surface. Moreover, oleaginous bases can prolong their topical residence time [122]. They are also inexpensive, easy to apply, and compatible with routine wound-care practice. However, future studies are needed to systematically examine formulation-related parameters of ointment-based AgNP formulations, including dispersion, release profiles, dose uniformity, aggregation, rheology, and stability under defined storage conditions.

Overall, the various formulations described above enhance the clinical relevance and practical viability of plant-mediated AgNPs for wound-healing applications. However, formulation can also complicate the attribution of wound-healing outcomes to plant-mediated AgNPs themselves. The polymer matrices, hydrogel networks, ointment bases, and dressing scaffolds may exert their own biological effects on wound closure, antimicrobial efficacy, angiogenesis, or the modulation of inflammation. Thus, a nanoparticle-free delivery control should be included and statistically compared with the complete AgNP-loaded formulation. Approximately half of the wound-healing AgNP formulation studies reviewed here clearly included nanoparticle-free vehicle controls in the wound-healing experiments.

### 4.2. Experimental Wound-Healing Models

Among the reviewed studies, three main categories of wound-healing models were identified: in vitro, ex vivo, and in vivo models. These three categories, along with examples, advantages, and disadvantages, are summarized in Table 4. In addition, the experimental wound-healing models and outcomes reported in each reviewed article are presented in Table 3. Although these models provide information for evaluating the wound-healing potential of plant-mediated AgNPs, each category has inherent limitations. A key limitation is their inability to fully recapitulate the complexity of the wound microenvironment. This limitation is particularly evident in conventional in vitro scratch assays, which are primarily used to assess collective cell migration as one component of the wound-healing process. However, these assays fail to capture the key biochemical mediators, complex enzymatic cascades, and interacting physiological and pathological features present in the actual wound microenvironment [123].

Attempts have been made to replicate some of these pathological or biochemical features by developing three-dimensional (3D) wound models, including reconstructed human skin equivalents and scaffold-based wound models [124]. Notably, none of the included studies tested plant-mediated AgNPs using a 3D wound-healing platform. However, such models have been used in other studies to evaluate the wound-healing potential of AgNP-based systems. For instance, Truzzi et al. used 3D reconstructed human skin equivalents to screen a topical vitamin D2–colloidal AgNP cream [125].

In addition to classical experimental methodologies, researchers have developed in silico models to mimic the wound environment. Interestingly, these platforms have been used to predict cellular, inflammatory, angiogenic, and tissue-remodeling responses in wounds. However, the predictive abilities of these models depend directly on the quality of the input data and experimental validation [126].

**Table 4:** Experimental wound-healing models used in the reviewed studies. The advantages and limitations of in vitro, ex vivo, and in vivo wound-healing models were summarized based on previous methodological reviews and wound-healing model comparisons [127,128,129]. Abbreviations: ECM, extracellular matrix; *S. aureus*, *Staphylococcus aureus*; *E. coli*, *Escherichia coli*; *K. pneumoniae*, *Klebsiella pneumoniae*.

Model Category	What It Mainly Assesses	Representative Examples	Pros	Cons
In vitro models	Cell migration, proliferation, and viability Preliminary wound closure Angiogenesis-related cellular responses	Scratch assays (fibroblast, keratinocyte, endothelial cell) Endothelial cell tube formation assays Wound-related enzyme inhibition assays in cells	Simple Rapid Low cost Early screening Reduces animal use	Does not represent full wound biological elements (systemic immune and vascular response, ECM remodeling, exudate dynamics) Limited prediction of in vivo healing
Ex vivo models	Wound microenvironment modulation Enzyme activity in wound-derived samples	Diabetic foot ulcer exudate assays MPO and collagenase inhibition in exudates	More biologically relevant than in vitro Bridges in vitro and in vivo testing	Lack of actual wound closure measurement Lacks systemic immune and vascular responses Limited standardization
In vivo models	Actual tissue repair Wound closure/contraction Re-epithelialization and granulation tissue formation Collagen remodeling Inflammation and angiogenesis Antibacterial performance in infected wounds	Various skin wound models including full-thickness excision, traumatic, burn, and diabetic wounds Infected wounds using *S. aureus*, MRSA, *E. coli*, or *K. pneumoniae* Oral and palatal mucosal wounds	Presence of systemic immune and vascular responses Enables histology and collagen assessment Suitable for more clinically relevant wounds like infected, diabetic, and burn wound settings	Higher cost and ethical burden Inter-study heterogeneity Rodent healing relies strongly on contraction rather than re-epithelialization and granulation tissue formation Direct clinical translation remains limited

### 4.3. Proposed Mechanisms Underlying the Wound-Healing Activity of AgNPs

**Figure 4:**
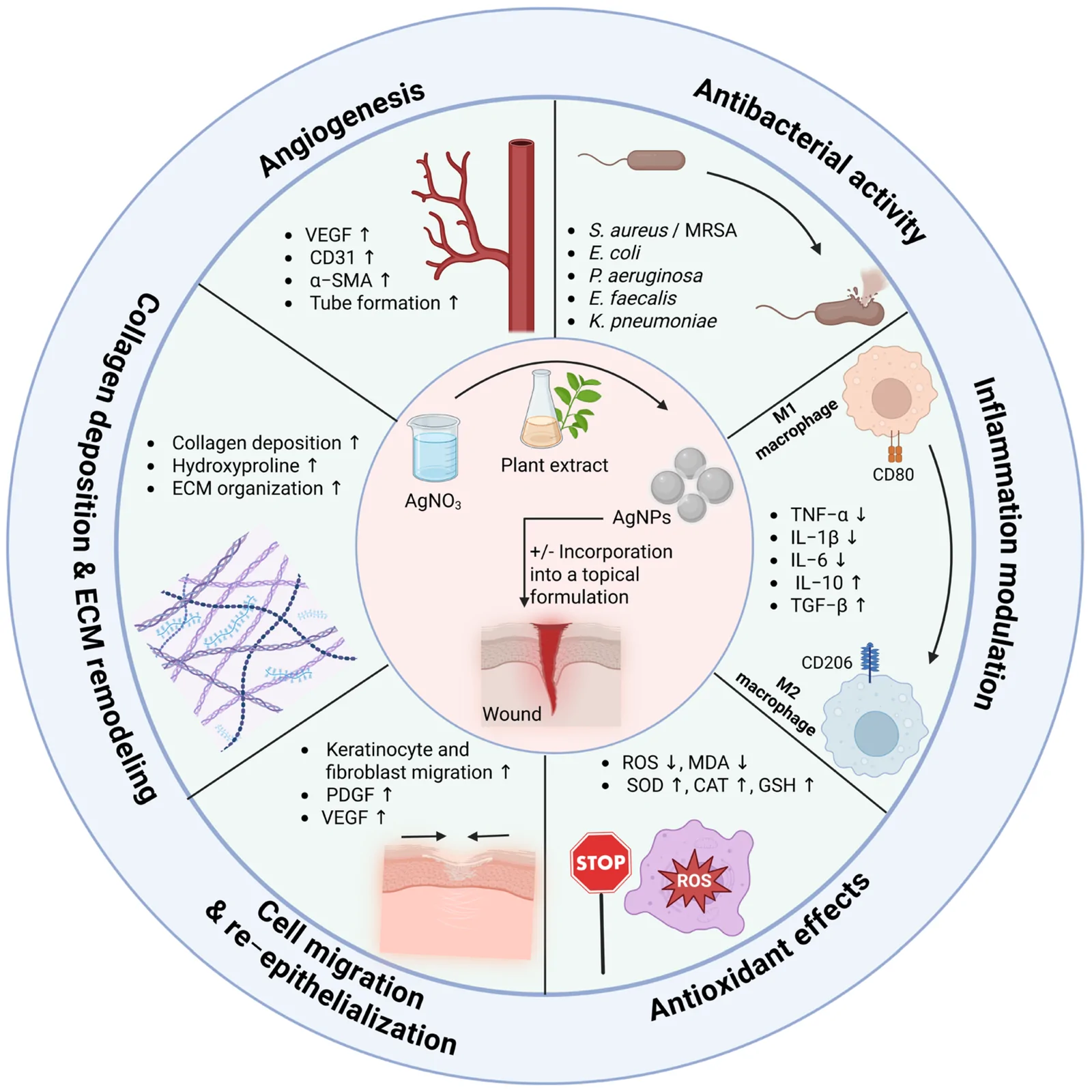
Proposed mechanisms of plant-mediated AgNPs in wound healing. Upward and downward arrows indicate increases and decreases, respectively. Abbreviations: *S. aureus*, *Staphylococcus aureus*; MRSA, methicillin-resistant *Staphylococcus aureus*; *E. coli*, *Escherichia coli*; *P. aeruginosa*, *Pseudomonas aeruginosa*; *E. faecalis*, *Enterococcus faecalis*; *K. pneumoniae*, *Klebsiella pneumoniae*; VEGF, vascular endothelial growth factor; PDGF, platelet-derived growth factor; TNF-α, tumor necrosis factor-alpha; IL, interleukin; TGF-β, transforming growth factor-beta; ROS, reactive oxygen species; MDA, malondialdehyde; SOD, superoxide dismutase; CAT, catalase; GSH, glutathione; ECM, extracellular matrix; α-SMA, alpha-smooth muscle actin. Created in BioRender. Billa, N. (2026) https://BioRender.com/wwkdone, accessed on 24 August 2026.

The proposed mechanisms underlying the wound-repair activity of plant-mediated AgNPs proposed across the reviewed articles included anti-inflammatory and antioxidant effects, enhanced cell proliferation, collagen deposition, extracellular matrix remodeling, and angiogenesis, as summarized below and in Figure 4.

#### 4.3.1. Antibacterial Activity

It is noteworthy that most studies provided evidence of the antibacterial activity of plant-mediated AgNPs. However, antibacterial activity is not, by itself, a direct wound-healing mechanism but rather supports wound healing by reducing the microbial burden in infected wounds. A lower bacterial load reduces local inflammation and tissue damage caused by bacterial infection. This creates a more favorable environment for tissue regeneration [130].

Antibacterial activity was usually assessed using agar diffusion, growth inhibition, and antibiofilm assays. Two studies directly measured bacterial levels in infected wound tissue [19,61]. Although in vitro antibacterial and antibiofilm assays provide useful proof-of-concept evidence, infected wound models are more clinically relevant because they can determine whether AgNPs reduce infection when applied to the wound in their final formulation. Only a limited number of studies focused on elucidating the mechanisms underlying antibacterial activity. For example, Chang et al. incorporated *Citrus aurantium*-mediated AgNPs into a citrus pectin hydrogel that was shown to damage bacterial membranes, induce ATP depletion, promote leakage of proteins and DNA, and inhibit biofilm formation [61]. Ahmad et al. showed that *Coriandrum sativum*-mediated AgNPs disrupted bacterial membranes, generated reactive oxygen species, caused adenosine triphosphate (ATP) depletion, promoted leakage of DNA, RNA, and proteins, and inhibited biofilm formation [19]. Similarly, in the *Azadirachta indica*-mediated AgNP hydrogel study, SEM analysis showed surface damage to *E. coli* cells [78].

#### 4.3.2. Anti-Inflammatory Activity

Attenuation of inflammation was one of the most frequently proposed mechanisms underlying the wound-healing activity of plant-mediated AgNPs. Histological evidence of reduced inflammation, such as decreased inflammatory-cell infiltration in hematoxylin and eosin (H&E)-stained wound tissue, was frequently reported (Table 3). In addition, several groups provided molecular or cellular evidence supporting anti-inflammatory effects. Guzzatti et al. reported that AgNPs synthesized using *Curcuma longa* and *Euterpe oleracea* and formulated into hydrogels reduced the inflammatory infiltrate in rat palatal wound tissue. The Curcuma-mediated AgNP hydrogel was also shown to reduce tumor necrosis factor-α (TNF-α) and interleukin-1β (IL-1β) while increasing the expression of interleukin-4 (IL-4) and transforming growth factor-β (TGF-β) [58]. Chang et al. found that *Citrus aurantium*-mediated AgNPs incorporated into a citrus pectin hydrogel reduced TNF-α expression, and increased IL-10 expression. In addition, this AgNP-loaded hydrogel shifted macrophage marker expression from CD80-positive M1-like markers toward CD206-positive M2-like markers in MRSA-infected diabetic wounds [61]. Wang et al. supported the anti-inflammatory effects of an *Aronia melanocarpa*-based AgNP hydrogel using transcriptomic analysis, showing that treated MRSA-infected wounds had reduced expression of interleukin-17 (IL-17)-related inflammatory genes, including C-X-C motif chemokine ligand 1 (CXCL1), CXCL2, granulocyte colony-stimulating factor (G-CSF), thymus- and activation-regulated chemokine (TARC), and eotaxin [53]. Govindarasu et al. demonstrated that *Lannea coromandelica*-mediated AgNPs encapsulated in chitosan downregulated interleukin-6 (IL-6) and TNF-α and upregulated TGF-β and vascular endothelial growth factor (VEGF). These findings suggest that this formulation may help promote the transition from the inflammatory phase to the proliferative phase of wound healing [59].

#### 4.3.3. Antioxidant Activity

Another commonly proposed mechanism underlying the wound-repair activity of plant-mediated AgNPs was their antioxidant effect, which was evaluated using chemical assays such as 2,2-diphenyl-1-picrylhydrazyl (DPPH) and 2,2′-azino-bis(3-ethylbenzothiazoline-6-sulfonic acid) (ABTS) radical-scavenging assays, hydrogen peroxide scavenging assays, and the ferric reducing antioxidant power (FRAP) assay. Only a few studies directly measured oxidative stress levels within wound tissues. For the *Curcuma longa*- and *Euterpe oleracea*-mediated AgNP hydrogels, Guzzatti et al. reported modulation of oxidative and nitrosative stress in rat palatal wounds, as shown by reduced 2′,7′-dichlorofluorescein (DCF) fluorescence and nitrite levels in tissue samples [58]. Chang et al. found that a *Citrus aurantium*-mediated AgNP hydrogel exhibited antioxidant capacity and limited reactive oxygen species (ROS) generation in RAW264.7 macrophages [61].

#### 4.3.4. Cell Migration, Proliferation, and Re-Epithelialization

Three studies included in this review proposed that the wound-healing properties of plant-mediated AgNPs may be related to the promotion of cell migration, proliferation, and re-epithelialization. These functions were mainly assessed using in vitro scratch assays in fibroblasts, keratinocytes, or endothelial cells. For instance, plant-derived AgNPs from *Euphorbia hirta*, *Ocimum americanum*, *Nigella sativa*, *Cyperus rotundus*, *Actinidia deliciosa*, *Rhizophora apiculata* and *Scutellaria barbata* enhanced cell migration in wound scratch assays [48,55,56,70,73,74]. Mechanistic studies were less commonly performed. For instance, Palanisamy et al. demonstrated that *Nigella sativa*-mediated AgNPs promoted HaCaT keratinocyte scratch closure, accompanied by the upregulation of platelet-derived growth factor (PDGF) and VEGF [56]. This suggests a possible role for growth factor signaling pathways in AgNP-associated enhancement of cell migration. Transcriptomic analysis performed by Wang et al. revealed that an *Aronia melanocarpa*-based AgNP hydrogel promoted the expression of genes associated with epithelial adhesion and repressed genes associated with inflammatory signaling [53].

#### 4.3.5. Collagen Deposition and Extracellular Matrix Remodeling

Increased collagen deposition and ECM remodeling were commonly documented in plant-mediated AgNP in vivo wound-healing studies. These observations were mainly assessed using H&E and Masson’s trichrome staining. Using these staining approaches, the studies demonstrated greater collagen deposition, more organized collagen fibers, improved granulation tissue formation, and improved dermal reconstruction compared with controls. For instance, Wang et al. showed that an *Aronia melanocarpa*-based AgNP hydrogel enhanced collagen deposition in MRSA-infected wounds, along with increased re-epithelialization [53]. Chang et al. reported similar results with a *Citrus aurantium*-mediated AgNP hydrogel, which promoted collagen deposition and tissue remodeling in MRSA-infected diabetic wounds [61]. Ahmad et al. reported that treatment with a topical gel containing *Camellia sinensis*-mediated AgNPs resulted in thicker and more organized collagen bundles in infected wounds [67]. Fatima et al. demonstrated improved dermal architecture and collagen organization with a *Tridax procumbens*-mediated AgNP gel in an excision wound model [77]. Guzzatti et al. showed that *Curcuma longa*-mediated AgNPs increased the collagen area in rat palatal wounds [58]. Many other groups reported increased collagen deposition, granulation tissue formation, and improved dermal remodeling in in vivo studies with AgNPs synthesized using *Punica granatum*, *Dendrobium officinale*, and *Azadirachta indica* [52,57,78]. However, in most cases, ECM remodeling was inferred primarily from histological analysis of collagen fibers rather than from direct investigation of the molecular pathways regulating ECM remodeling.

#### 4.3.6. Angiogenesis

Angiogenesis was another proposed mechanism contributing to AgNP-mediated wound repair. Collectively, the reviewed evidence suggests that angiogenesis may contribute to the wound-healing effects of selected plant-mediated AgNP systems. In many of the aforementioned in vivo studies, the assessment of angiogenesis was limited to histological evidence of neovascularization or vascularized granulation tissue (Table 3). More direct evidence was provided by studies that measured endothelial responses or angiogenesis markers. For example, Chang et al. found that a *Citrus aurantium*-mediated AgNP hydrogel significantly promoted HUVEC migration and tube formation in vitro. They also demonstrated increased expression of VEGF, CD31, and Ki67 in MRSA-infected diabetic wounds, suggesting enhanced endothelial activation, neovascularization, and proliferative wound repair [61]. Ahmad et al. found that *Coriandrum sativum*-mediated AgNPs promoted the expression of CD31 and VEGF in infected wounds [19]. Finally, Wang et al. demonstrated that the *Aronia melanocarpa*-based AgNP hydrogel promoted vascular remodeling in MRSA-infected wounds, with CD31 staining suggesting improved neovascularization [53].

## 5. Cross-Study Evaluation of Process–Property–Performance Relationships

The process–property–performance (PPP) framework is supported by broader evidence that plant-mediated synthesis conditions affect AgNP properties and that these properties can influence biological responses [10,25,26,27,28]. However, our screening of the 34 included publications showed that all stages represented in this framework (Figure 1) were rarely examined together within a single study. We therefore cross-tabulated the publications at the study level to determine how often each relationship was directly assessed (Table 5).

For this analysis, the process category was restricted to the plant-mediated synthesis variables presented in Figure 1: plant species, plant part, extraction solvent, AgNO_3_ concentration, pH, temperature, reaction time, and extract-to-AgNO_3_ ratio. Comparisons between green and chemically synthesized AgNPs, formulation composition, administered AgNP dose, and conditions of use were excluded because they were outside this predefined set of process variables. A relationship was counted only when an eligible upstream variable was compared at two or more levels, and an eligible downstream variable was measured within the same study. Direct comparisons with limited statistical analysis were retained but distinguished from formally supported evidence.

Fourteen publications directly assessed at least one process-to-property relationship [48,50,51,52,53,54,57,58,61,64,67,68,71,76]. Seven of these relied only on UV–Vis/SPR responses [50,51,57,61,64,68,76], whereas seven also examined at least one core physicochemical property, including size, morphology, PDI, or zeta potential [48,52,53,54,58,67,71]. Most used OFAT or descriptive comparisons, and only Karthikeyan et al. used a formal RSM model to test both main and interaction effects [48]. In contrast, only two publications directly assessed a process-to-performance relationship by comparing AgNP systems prepared from different botanical sources [54,58]. These comparisons provided some direct evidence, but attribution to the plant source remained limited because several material characteristics varied together and direct pairwise statistical analyses were incomplete. No publication directly tested whether differences in size, morphology, PDI, zeta potential, colloidal stability, Ag^+^ release, or optical response accounted for differences in therapeutic performance. Therefore, none of the included publications established a complete process-to-property-to-performance chain. Thus, Figure 1 presents relationships supported by the broader field, whereas the present screening shows that these relationships have not yet been tested systematically in the included wound-healing literature.

## 6. Challenges and Future Perspectives

In recent years, plant-mediated AgNPs have gained considerable attention as candidates for wound-healing applications. This interest may reflect the combined biological activities of silver nanoparticles and plant-derived compounds, together with advances in wound-delivery technologies. However, a number of challenges limit their clinical translation and large-scale production. Addressing these challenges will be critical for translating plant-mediated AgNPs from experimental systems into clinically relevant wound-care technologies.

### 6.1. Batch-to-Batch Reproducibility

One of the major limitations hindering the scale-up and subsequent clinical translation of plant-mediated AgNPs is limited production reproducibility. Reproducibility is a general challenge for all nanoformulations, but this challenge is particularly pronounced for plant-mediated AgNPs because of the heterogeneity of the plant-derived components used in their production. Batch-to-batch variability in synthesis yield and physicochemical characteristics has been reported for AgNPs synthesized using plants [81]. The production and metabolism of phytochemicals responsible for the reduction and capping of metal nanoparticles may be influenced by the plant source, light exposure, soil composition and nutrient content, water availability, harvesting time, and plant age [35]. To illustrate this point, extracts prepared from *Paullinia cupana* leaves collected during the dry and rainy seasons yielded AgNPs that differed in SPR peak intensity, hydrodynamic size, size distribution, particle concentration, and biological activity. The authors attributed these differences to season-dependent changes in the secondary metabolite composition of the extracts [36].

One approach to mitigate this limitation is to standardize the botanical source and harvest time. Each extract batch should be characterized using consistent phytochemical measurements, such as TPC and TFC assays, chromatographic fingerprinting, and, where possible, quantification of relevant marker compounds. Extraction yield and extract concentration should also be reported on a dry-mass basis to support batch-to-batch comparisons. Greenhouse cultivation can provide controlled growth conditions and thereby reduce batch-to-batch variability in plant phytochemical profiles.

Another practical measure to address reproducibility is to use purified molecules from plant extracts, such as quercetin, gallic acid, and tannic acid, instead of crude extracts [131,132,133]. In this approach, a standardized amount of a purified phytochemical or a defined combination of phytochemicals may be used for green synthesis, as demonstrated by Jain and Mehata, who synthesized AgNPs using *Ocimum sanctum* leaf extract and compared them directly with AgNPs synthesized using purified quercetin, a major flavonoid present in that extract. The results showed that AgNPs synthesized using purified quercetin had optical, morphological, and antibacterial properties very similar to those of extract-mediated AgNPs. However, purified quercetin mediated faster reduction and produced slightly smaller nanoparticles with higher short-term colloidal stability [134]. These findings suggest that purified phytochemicals may improve production consistency by providing a more defined reducing and capping system, although direct batch-to-batch reproducibility studies are still needed. Although this strategy addresses reproducibility concerns, the purification and concentration of phytochemicals from plant extracts add further steps that may require the use of additional chemicals and energy inputs, thereby undermining the principles of green synthesis [135].

### 6.2. Incomplete Reporting of Synthesis Parameters

Another challenge identified across the reviewed studies was the incomplete reporting of reaction parameters. Only one study reported all key variables, including AgNO_3_ concentration, the extract-to-AgNO_3_ ratio, pH, temperature, stirring speed, and reaction time. The most frequently missing parameters were pH and stirring speed during synthesis (Table 1). As discussed above, reproducibility challenges arise mainly from the variability among natural sources. However, this concern can be partially addressed through comprehensive control of reaction parameters and their precise reporting in publications. Therefore, we recommend that researchers consider adopting a unified methodological reporting framework for the green synthesis of metallic nanoparticles, in which, at a minimum, all of the aforementioned synthesis parameters are presented.

### 6.3. Spectroscopy-Driven Optimization

Another challenge identified was that optimization of plant-mediated AgNP synthesis was often based primarily on UV–Vis spectra and SPR characteristics rather than on nanoparticle physicochemical properties or wound-relevant performance outcomes (Table 2). Although spectroscopic analysis is useful for rapid preliminary screening of AgNP formation, it is insufficient as the sole optimization endpoint for wound-healing applications. In this review, we identified several examples in which reaction conditions that increased SPR intensity were not selected as the final optimization conditions because the resulting AgNPs exhibited precipitation or aggregation, excessive particle size, or biological incompatibility [57,61,67]. In nearly all the reviewed cases, biological performance-based optimization was not conducted. Moreover, biological assays were usually performed only after the optimized AgNPs had been selected, rather than being used as endpoints during the optimization process. Future optimization studies should not rely solely on SPR quality. Although SPR features are useful, it is more appropriate to consider them as only one component of AgNP optimization.

Physicochemical attributes such as particle size, PDI, zeta potential, morphology, colloidal stability, and Ag^+^ release should be evaluated in parallel and used as potential optimization endpoints because these characteristics can directly or indirectly affect the biological performance of synthesized AgNPs [10]. Reporting of these characterization variables was uneven. With the publication as the unit of analysis, 33 of the 34 publications reported particle size and 33 reported morphology, whereas only 12 reported PDI and 20 reported zeta potential (Table 1). Thus, particle-size distribution and surface charge were not reported in a substantial proportion of the publications, limiting the assessment of colloidal behavior and comparisons among AgNP systems.

### 6.4. AgNP Stability and Compatibility Within Formulations

Another formulation-related limitation was the incomplete understanding of how wound-care delivery vehicles affect the stability of plant-mediated AgNPs. Incorporating AgNPs into polymeric networks may induce changes in nanoparticle behavior. Excipients such as polymers, crosslinkers, oils, surfactants, salts, and pH modifiers may interact with nanoparticles and alter their surface charge [136], thereby influencing aggregation, AgNP dissolution, and release kinetics [137]. For example, Alaroud et al. demonstrated that although AgNP-loaded Carbopol and Pluronic F127 hydrogels both remained stable during storage, they exhibited matrix-specific release profiles upon contact with agar. These findings suggest that the behavior of AgNPs within delivery vehicles depends on both the polymer matrix and the surrounding release environment [138]. In many studies, AgNPs were synthesized and characterized as aqueous dispersions or dried nanoparticles before being incorporated into different vehicles. However, these studies often did not evaluate nanoparticle compatibility with other formulation components.

Several of the reviewed articles evaluated formulation-related properties such as pH, viscosity, spreadability, swelling, water retention, and rheology [67,72,77], but these measurements did not usually establish whether the AgNPs remained stable within the final product. This limitation was reflected in the extracted data. Six publications included some assessment of colloidal, storage, or short-term physical stability, and two additional publications examined biodegradation (Table S2). Among the six stability studies, three evaluated the final wound formulation, and only one monitored it for three months under defined storage conditions.

Release behavior was also rarely examined. Four publications conducted release experiments (Table S1); one quantified Ag^+^ by inductively coupled plasma–optical emission spectrometry (ICP–OES), whereas the other three used UV–Vis spectroscopy and reported AgNP release without distinguishing among intact nanoparticles, dissolved Ag^+^, and total silver. These gaps make it difficult to assess long-term formulation stability and the form and amount of silver released at the wound site. Future studies should therefore assess AgNP–formulation compatibility as a distinct quality attribute by monitoring changes in particle size, PDI, and zeta potential after incorporation, aggregation within the delivery vehicle, AgNP and Ag^+^ release kinetics, and long-term storage stability.

### 6.5. Limited Evidence for the Contribution of the Phytochemical Corona

Although several studies included in this review attributed part of the biological activity of plant-mediated AgNPs to their phytochemical corona [15,17,18,19], this contribution was rarely tested using controls that could separate it from the effects of silver or other nanoparticle properties. Comparison with the plant extract alone can show whether the complete AgNP system performs better than the free extract, while comparison with chemically synthesized AgNPs that are physicochemically comparable can help assess the possible contribution of the plant-mediated synthesis route and surface chemistry.

Of the 34 included publications, seven used an extract-only control (Table 3), and the AgNP systems generally showed more favorable wound-related activity than the corresponding extracts [46,50,56,61,62,65,73]. Only two publications included chemically synthesized AgNPs (Table 3), and both reported more favorable outcomes for the green AgNP system [19,59]. However, none included both controls simultaneously. Furthermore, the green and chemical AgNPs in Ahmad et al. differed markedly in hydrodynamic size [19], whereas in Govindarasu et al., the particle-size ranges overlapped but the green system also contained chitosan [59]. These comparisons indicate greater activity of the complete green AgNP system but do not establish that the difference was caused specifically by the phytochemical corona. Future studies should include both controls and match the green and chemical AgNPs as closely as possible with respect to size, dose, surface charge, and other relevant properties.

### 6.6. Cytocompatibility, Dose Reporting and a Poorly Defined Therapeutic Window

Alongside efficacy, cytocompatibility must be assessed to determine whether an AgNP system has a suitable safety margin for wound application. However, such testing was reported in only 16 of the 34 publications (Table S3). Thirteen of these used wound-relevant fibroblasts or keratinocytes: 12 used fibroblasts and only one used keratinocytes. The remaining three studies used lymphocytes, embryonic kidney cells, or endothelial cells. Exposure periods were generally short, and the tested doses and reporting formats varied considerably. Therefore, reports that individual systems were cytocompatible at selected doses do not define a consistent safety margin for wound-relevant skin cells. Evidence based on other safety endpoints was also limited; the reported hemocompatibility, dermal irritation, and in vivo safety assessments are summarized in Table S3. Together with inconsistent dose reporting, these gaps make the therapeutic window difficult to define.

Clinically relevant dose ranges for plant-mediated AgNPs in wound-healing applications have not yet been well established. Although AgNPs may exert antimicrobial and pro-healing effects, these therapeutic benefits are dose-dependent; at higher doses or with prolonged exposure, they may be outweighed by cytotoxicity, oxidative stress, hemolysis, inhibition of fibroblast and keratinocyte migration, and delayed tissue regeneration [139].

Dose reporting varied substantially across the studies included in this review (Table 3). AgNP doses were expressed in heterogeneous units and formats, including concentrations in μg/mL, total nanoparticle mass in mg, and % w/w, while some studies did not report the administered dose. This inconsistency complicates comparisons among studies and makes it difficult to determine whether observed differences are attributable to nanoparticle physicochemical properties, formulation design, plant-derived capping agents, or simply differences in silver exposure. Future studies should report both the formulation concentration and the AgNP dose or silver-equivalent dose. For in vivo wound studies, the administered dose should preferably be normalized to wound area and reported together with the amount applied, frequency, and duration. Dose–response studies are also needed to define a dose range with an acceptable efficacy–safety balance [24].

### 6.7. Outcome Direction, Active Comparators, and Risk of Bias

All 34 included studies (34/34) reported at least one favorable wound-healing or wound-healing-related outcome for the plant-mediated AgNP system evaluated (Table 3). The only apparent exceptions were unchanged glycated hemoglobin (HbA1c) in one study and impaired scratch closure with matrix-free AgNPs in another [54,60]; neither contradicted the overall favorable outcome reported for the main system. However, this 100% success rate characterizes the available publication record rather than the intrinsic efficacy of these materials. It should also be viewed in light of the targeted scope of this review, which focused on studies evaluating plant-mediated AgNPs in wound-healing-related models. Thus, the favorable direction of the findings alone cannot establish therapeutic promise. To allow a more critical assessment, Table 3 now reports, for each in vivo publication, randomization, blinding of wound and histological assessments, group size, histology type, and statistical analysis of wound and histological outcomes.

Sample size affects the power and precision of an animal study and should be justified according to the expected effect size, variability, study design, significance level, and desired power, while avoiding unnecessary animal use [140]. Of the 34 included studies, 24 included in vivo evaluations. Two of these did not report group size. Among the remaining 22 studies, 21 parallel-group studies used 3–12 animals per group (median, 6), while one within-animal study used six rats in total. No a priori sample-size calculation or other prospective justification was identified. Therefore, the adequacy of these group sizes for estimating treatment efficacy remains uncertain.

Histology complements wound-closure measurements by showing the quality of tissue repair. Of the 24 in vivo publications, 19 included histology and five did not. Histological assessment was exclusively qualitative in nine studies, exclusively semi-quantitative in three, exclusively quantitative in one, and mixed in six. Qualitative assessment describes tissue morphology, semi-quantitative assessment uses ordinal scores, and quantitative assessment provides numerical measurements. Mixed approaches using validated scoring or morphometry generally provide stronger evidence than qualitative description alone, although their reliability still depends on standardized sampling, blinded assessment, animal-level replication, and appropriate statistics [141].

Randomization reduces selection bias, while blinding limits the influence of investigators’ expectations on outcome assessment [140,142]. Randomization was reported in only 9 of the 24 in vivo publications. None reported blinded assessment of wound outcomes, and only 2 of the 19 studies with histology reported blinded histological assessment. Although these procedures may have been performed but not reported, the corresponding risks of bias remain unclear.

Appropriate statistical analysis is essential for distinguishing treatment effects from random variation and for estimating their magnitude and precision. Inferential analysis of wound outcomes was reported in 20 studies; three did not report such analysis, and one provided descriptive results only. Among the ten studies using quantitative, semi-quantitative, or mixed histology, only five reported inferential histological analysis. Common statistical limitations included unclear handling of repeated or clustered measurements; unspecified endpoint-specific tests, post hoc comparisons, or multiplicity adjustments; poorly defined experimental units; and incomplete reporting of variability, exact *p* values, effect sizes, and confidence intervals. Consequently, the frequent reporting of statistical significance does not necessarily indicate appropriate analysis, and the available results may not provide precise or robust estimates of wound-healing efficacy.

Alongside the risk-of-bias assessment, active comparators provide an important benchmark for judging whether the favorable outcomes reported for plant-mediated AgNP systems represent an improvement over existing treatments. Only 13 of the 34 publications included an active comparator (Table 3). Silver sulfadiazine was used most often (four publications), followed by povidone-iodine or Betadine (three publications). In nine publications, the AgNP system showed at least some numerical advantage or was reported to be advantageous [49,53,58,63,64,67,68,75,77]; in three, its performance was broadly similar or slightly lower [51,55,71]; and in one, wound closure was lower than with the comparator [54]. However, only three publications clearly reported a statistically significant benefit over the comparator [49,63,77], while one showed a significant benefit at early time points but not at the final assessment [53]. Most studies did not report adequate direct pairwise comparisons, and none used a formal equivalence or non-inferiority design. Therefore, it remains uncertain whether plant-mediated AgNP systems consistently provide better outcomes than established wound treatments.

Overall, these 34 studies provide early evidence that plant-mediated AgNPs may improve wound healing, but they do not conclusively establish efficacy, clinical promise, or an advantage over existing treatments. Confidence in the findings is limited by the lack of prospective sample-size justification, limited reporting of randomization and blinding, weaknesses in histological and statistical analyses, and the small number of well-analyzed active-comparator studies. Future animal studies should justify sample size, define primary outcomes, use randomization and blinded assessment, and include suitable controls and clinically relevant active comparators. When an active comparator is used, direct comparisons should be planned and supported by appropriate statistical analyses. Standardized outcome measures, effect sizes, confidence intervals, and negative or non-significant findings should also be reported. Independent studies using clinically relevant wound models are needed before firm conclusions can be drawn.

### 6.8. Regulatory Considerations for Clinical Translation

Regulatory requirements for plant-mediated AgNP wound therapies depend on the jurisdiction and vary according to the intended use and mode of action of the final product. A gel, dressing, or other wound-care system may therefore follow a drug, medical device, or combination product pathway [143]. Regulatory assessment should address the finished product rather than the isolated AgNPs alone. This requires reproducible manufacturing and control of composition, particle-size distribution, surface properties, aggregation, impurities, Ag^+^ release, and stability, together with evidence of safety and efficacy or performance for the intended use [144]. For wound-contacting devices, biological evaluation should reflect the nature and duration of tissue contact within a risk-management framework [145]. In the European Union, devices containing nanomaterials are also classified according to their potential for internal exposure [146]. The characterization, release, stability, and safety gaps identified in this review would therefore need to be addressed before regulatory approval and clinical translation.

## 7. Conclusions

Overall, the available studies suggest that plant-mediated AgNP systems may support wound healing; however, the evidence remains preliminary because of variations in study design and limited reporting and control of key sources of bias. Nanotechnology nevertheless offers a versatile platform for the localized delivery and controlled release of silver species in wound-care systems. In this context, AgNPs are not merely generic green products but process-dependent materials whose biological activity is shaped by the conditions under which they are synthesized. However, these conditions are not fully standardized across studies; neither are the therapeutic doses nor the biological models used to evaluate wound-repair outcomes.

Most studies reported favorable effects on wound closure, bacterial burden, inflammation, oxidative stress, collagen deposition, re-epithelialization, and angiogenesis; however, systematic integration and interpretation of these findings remain challenging because of heterogeneity in AgNP systems, formulations, administered doses, and wound models. In addition to this heterogeneity, active comparators were included in only a limited proportion of the reviewed publications, and the available head-to-head evidence was insufficient to establish a consistent advantage over established, inexpensive wound treatments. This limits assessment of the added value of plant-mediated AgNPs for clinical translation and highlights the need for better-designed studies using clinically relevant comparators and appropriate direct statistical comparisons.

Furthermore, plant-mediated AgNPs are often optimized primarily on the basis of instrumental measurements, particularly UV–Vis spectroscopy and associated SPR characteristics, whereas physicochemical quality attributes such as particle size, PDI, zeta potential, morphology, stability, and Ag^+^ release kinetics are typically evaluated only after the optimal synthesis conditions have been selected. In addition, cytocompatibility and wound-relevant biological endpoints are generally assessed only for the final optimized AgNP system. Thus, synthesis conditions were often selected mainly on the basis of spectroscopic features that do not necessarily predict wound-healing performance.

Additionally, while numerous studies incorporated AgNPs into hydrogels, topical gels, dressings, ointments, or polymeric matrices, the impact of the delivery vehicle on AgNP aggregation, surface charge, release kinetics, stability, and biological activity was rarely evaluated.

Although the reviewed studies generally reported favorable wound-healing outcomes, the underlying molecular mechanisms remain insufficiently characterized. Direct evidence regarding macrophage polarization, cytokine regulation, oxidative-stress signaling, fibroblast and keratinocyte responses, angiogenic pathway activation, and ECM-remodeling mechanisms is particularly scarce. Attributing these mechanisms and biological effects specifically to the phytochemical corona also remains difficult. Although comparisons with extract-only and chemically synthesized AgNP controls generally favored the green AgNP systems, no study included both controls, and the comparator nanoparticles were not adequately matched in their physicochemical properties.

Thus, this review positions plant-mediated AgNP wound-healing systems within a process–property–performance framework and emphasizes the need for rigorous, standardized evaluation and reporting at each stage. Adoption of this framework may support the translation of experimental findings into clinically relevant wound-care technologies.

## Figures and Tables

**Table 2:** Optimization strategies used for plant-mediated synthesis of AgNPs intended for wound-healing applications. Abbreviations: AgNPs, silver nanoparticles; RSM, response surface methodology; OFAT, one-factor-at-a-time; SPR, surface plasmon resonance.

Plant Species Used for AgNP Synthesis	Optimization Method	Optimized Synthesis Factors	Optimal Synthesis Condition	Optimization Endpoint (Selection Criterion)	Ref.
*Euphorbia hirta*; *Ocimum americanum*	RSM	AgNO_3_ concentration	1.03 mM	Particle size	[48]
		Extract to AgNO_3_ ratio	24.75:1 *v*/*v*		
*Punica granatum*	OFAT	AgNO_3_ concentration	2 mM	SPR spectra	[52]
		Initial reaction time	15 min		
*Dendrobium officinale*	OFAT	Extract concentration	15 mg/mL	SPR spectra	[57]
		AgNO_3_ concentration	60 mM		
		pH	7		
		Temperature	50 °C		
		Reaction time	7 h		
*Citrus aurantium* L.	OFAT	AgNO_3_ concentration	5 mM	SPR spectra	[61]
		Extract to AgNO_3_ ratio	1:9 *v*/*v*		
		pH	10		
		Temperature	90 °C		
		Reaction time	1 h		
*Camellia sinensis*	OFAT	AgNO_3_ concentration	6 mM	SPR spectra	[67]
		Extract to AgNO_3_ ratio	1:9 *v*/*v*		
		Temperature	40 °C		
		Reaction time	6 h		
*Prunus avium* L.	OFAT	AgNO_3_ concentration	15 mM	SPR spectra	[51]
		Temperature	85 °C		
		Reaction time	45 min		
*Petroselinum crispum*	OFAT	AgNO_3_ concentration	15 mM	SPR spectra	[68]
		Temperature	85 °C		
		Reaction time	105 min		

**Table 3:** Overview of plant-mediated AgNP formulations, administered exposure, wound-healing models, controls, outcomes, and key in vivo risk-of-bias items in the reviewed studies. Upward and downward arrows indicate increases and decreases, respectively. Abbreviations: NA, not applicable; NR, not reported; NC, negative or untreated control; VC, vehicle or AgNP-free formulation control; PE, plant-extract control; BFC, base-formulation control; AgNP-only, AgNPs tested without the accompanying delivery formulation; CS-AgNP, chemically synthesized AgNP comparator; PC, positive control; AC, active comparator; R, randomization; B-W, blinding of wound-outcome assessment; B-H, blinding of histological assessment; n, group sample size; H, histological assessment type; S-W, statistical analysis of wound outcomes; S-H, statistical analysis of histological outcomes; qual., qualitative; quan., quantitative; semi-quan., semi-quantitative.

Plant Species Used for Green Synthesis of AgNPs	AgNP System Evaluated for Wound Healing	AgNPs Loading Dose in the Formulation	Wound-Healing Study Model(s)	AgNP Administered Exposure (Amount, Duration)	Wound-Healing Control and Comparator Group(s)	Outcome(s)	Key In Vivo Risk-of-Bias Items	Study Year	Ref.
*Pyrrosia petiolosa* (Christ) Ching	AgNPs without an added delivery material	NA	In vivo (full-thickness traumatic wound model in KM mice)	5 g/kg AgNP suspension ^1^, once daily for 21 days	NC PE	In vivo: ↑ wound closure; ↓ inflammation; ↑ neovascularization/granulation tissue; ↑ tissue repair	R: reported B-W: NR B-H: reported n = 7/group H: qual. S-W: reported S-H: NA	2026	[46]
*Pinus eldarica*	A two-layer wound dressing composed of an AgNPs-loaded PVA-alginate nanofiber layer and an alginate hydrogel layer loaded with ZIF-8	32 μg/mL	In vitro (HaCaT keratinocyte scratch assay) In vivo (full-thickness burn wound rat model)	In vitro: Amount NR, 48 h ^2^ In vivo: NR	NC BFC (AgNPs-loaded PVA-Alg layer)	In vitro: ↑ HaCaT migration In vivo: ↑ wound closure; ↓ neutrophilic infiltration; ↑ re-epithelialization; ↑ collagen deposition and tissue remodeling	R: NR B-W: NR B-H: NR n = 4/group H: semi-quan. S-W: NR S-H: NR	2026	[47]
*Euphorbia hirta*; *Ocimum americanum*	AgNPs without an added delivery material	NA	In vitro (3T3-L1 fibroblast scratch assay)	40 μg/mL AgNPs, 24 h	NC	In vitro: ↑ fibroblast migration and wound closure	NA	2026	[48]
*Hibiscus rosa-sinensis*; *Cymbopogon citratus*	AgNPs-loaded topical Carbopol gel	0.2% *w*/*v*	In vivo (full-thickness excision wound model in Swiss albino mice)	Amount NR, once daily until complete healing	NC AC (Megaheal^®^ colloidal-silver gel)	In vivo: ↑ wound closure; ↑ re-epithelialization; ↑ granulation tissue; ↑ collagen deposition	R: NR B-W: NR B-H: NR n = NR H: qual. S-W: reported S-H: NA	2026	[49]
*Cydonia oblonga* L.	AgNPs without an added delivery material	NA	In vitro (wound-healing-related enzyme inhibition assay for myeloperoxidase and collagenase)	Amount NR, 15 and 20 min	PE PC (quercetin; oleanolic acid)	In vitro: ↓ myeloperoxidase; ↓ collagenase	NA	2026	[50]
*Prunus avium* L.	AgNPs-loaded Vaseline ointment	1% *w*/*w*	In vivo (burn wound model in Wistar rats)	Amount NR, once daily for 14 days	NC VC AC (1% silver sulfadiazine)	In vivo: ↑ burn wound closure; ↓ potential infection and oxidative stress	R: reported B-W: NR B-H: NA n = 6/group ^3^ H: NA S-W: reported S-H: NA	2026	[51]
*Punica granatum*	AgNPs-loaded Carbopol hydrogel	1 mM	In vivo (full-thickness excision wound model in female mice)	0.2 g gel, once daily until epithelialization	NC VC	In vivo ^4^: ↑ wound closure; ↑ re-epithelialization; ↑ angiogenesis; ↑ fibroblast proliferation; ↑ collagen deposition; ↓ inflammation	R: reported B-W: NR B-H: reported n = 5/group H: semi-quan. S-W: reported S-H: NR	2026	[52]
*Aronia melanocarpa*	pH and hyaluronidase-responsive hyaluronic acid hydrogel containing in situ synthesized AgNPs	NR	In vivo (full-thickness MRSA-infected wound model in rats)	NR	NC VC AC (medical Ag^+^ wound dressing)	In vivo: ↑ infected wound healing; ↓ bacterial burden; ↓ inflammation; ↑ collagen deposition; ↑ angiogenesis; ↑ re-epithelialization	R: NR B-W: NR B-H: NR n = 3/group H: qual. + quan. S-W: reported S-H: reported	2025	[53]
*Tagetes erecta*; *Portulaca oleracea*	AgNPs-loaded topical Carbopol gel containing seven medicinal plant extracts	0.1% *w*/*w*	In vivo (STZ-induced diabetic excision wound model in Wistar rats)	Amount NR, twice daily until healing	NC AC (povidone iodine ointment)	In vivo: ↑ diabetic wound closure; ↓ bacterial load; ↑ epithelialization; HbA1c unchanged	R: NR B-W: NR B-H: NA n = 4/group H: NA S-W: reported S-H: NA	2025	[54]
*Cyperus rotundus*	AgNPs-loaded Carbopol hydrogel	10 mg ^5^	In vitro wound scratch assay (human dermal fibroblasts-HDF) In vivo (full-thickness excision wound model in Wistar rats)	NR	NC AC (mupirocin ointment)	In vitro: ↑ HDF migration and wound closure In vivo: ↑ wound contraction; ↑ re-epithelialization; ↑ hair follicle and skin regeneration; no skin irritation	R: NR B-W: NR B-H: NR n = 6/group H: qual. + quan. + semi-quan. S-W: reported S-H: descriptive only	2025	[55]
*Nigella sativa* L.	AgNPs without an added delivery material	NA	In vitro wound scratch assay (HaCaT keratinocytes)	Amount NR, 48 h	PE PC (allantoin 50 µg/mL)	In vitro: ↑ keratinocyte migration/wound closure; ↑ PDGF and VEGF expression	NA	2025	[56]
*Dendrobium officinale* Kimura et Migo	AgNPs without an added delivery material	NA	In vivo (full-thickness excision wound model in ICR mice)	200 µL AgNP suspension ^6^, every 3 days for 18 days	NC	In vivo: ↑ wound closure; ↑ neovascularization; ↑ collagen fiber reconstruction; ↑ skin appendage regeneration; ↓ scar width; ↓ inflammation	R: reported B-W: NR B-H: NR n = 8/group H: qual. + quan. S-W: reported S-H: descriptive only	2025	[57]
*Curcuma longa* L.	AgNPs-loaded hydrogel	0.125 mg/mL	In vivo (rat palatal mucosa wound model)	50 µL hydrogel, once daily for 5 days	NC AC (Omcilon^®^ oral paste; Photobiomodulation with a 660 nm laser)	In vivo: ↑ wound contraction; ↓ inflammatory infiltrate; ↑ collagen area; ↓ pro-inflammatory cytokines; ↑ anti-inflammatory cytokines; ↓ oxidative and nitrosative stress; better results by AgNPs-Curcuma vs. AgNPs-Açai	R: reported B-W: NR B-H: NR n = 12/group H: quan. S-W: reported S-H: reported	2025	[58]
*Euterpe oleracea*									
*Lannea coromandelica*	Chitosan-encapsulated AgNP system without a defined dosage form	NA	In vitro scratch assay (NIH/3T3 fibroblasts) In vivo (STZ/high-fat diet-induced diabetic full-thickness excision wound model in BALB/c mice)	In vitro: amount NR, 24 h In vivo: 100 µL AgNP suspension ^7^, treatment reported at days 3, 7, and 14	NC CS-AgNP	In vitro: ↑ fibroblast migration In vivo: ↑ diabetic wound closure; ↑ re-epithelialization; ↑ granulation and connective tissue formation; ↑ collagen deposition; ↑ angiogenesis and skin appendage regeneration; ↓ inflammation	R: NR B-W: NR B-H: NR n = 6/group H: qual. + quan. S-W: NR S-H: reported	2025	[59]
*Prunus spinosa*	Chitosan–modified styrenic polymer nanocomposite containing dispersed AgNPs; no defined dosage form	4%, 7%, and 10% *w*/*w*	In vitro wound scratch assay (L929 fibroblasts)	50 μg/mL AgNP nanocomposite, 24 h	NC VC AgNP-only	In vitro: ↑ L929 scratch closure; 4% Ag showed best wound-healing effect; AgNPs alone impaired closure at tested dose	NA	2025	[60]
*Citrus aurantium* L.	AgNPs-loaded citrus pectin hydrogel	50 μg/mL	In vitro scratch assay, and tube formation assay (HUVECs) In vivo (MRSA-infected diabetic full-thickness wound model in C57BL/6 mice)	In vitro: amount NR, 12 h In vivo: 100 µL hydrogel, treatment reported at days 1, 3, 5, 7, and 9	NC PE VC AgNP-only	In vitro: ↑ HUVEC migration and tube formation In vivo: ↑ infected diabetic wound closure; ↓ MRSA burden; ↓ inflammation and M1 macrophages; ↑ M2 macrophages; ↑ collagen deposition; ↑ angiogenesis; ↑ re-epithelialization and skin appendage regeneration	R: reported B-W: NR B-H: NR n = 5/group H: qual. + quan. S-W: reported S-H: reported	2025	[61]
*Cassia sericea*	AgNPs without an added delivery material	NA	In vitro wound scratch assay (L929 fibroblast cells)	Amount NR, 24 h	NC PE PC (ascorbic acid)	In vitro: ↑ L929 cell migration and wound closure	NA	2026	[62]
*Premna integrifolia* L.	AgNPs-loaded thermosensitive poloxamer hydrogel	5% and 10% *w*/*w*	In vivo (*S. aureus*-infected open wound model in Swiss albino mice)	Amount NR, once daily until healing	NC AC (povidone-iodine ointment 10%)	In vivo: ↑ infected wound closure; ↑ re-epithelialization; ↑ collagen deposition; ↑ connective tissue formation; ↓ inflammation and discharge; 10% formulation showed the best healing response	R: NR B-W: NR B-H: NR n = 6/group H: qual. S-W: reported S-H: NA	2024	[63]
*Colocasia esculenta*	AgNPs-loaded film bandage	NR	In vivo (rabbit excision wound model)	NR	NC AC (BAND-AID^®^ HYDRO SEAL™)	In vivo: ↑ wound closure; ↑ collagen content	R: NR B-W: NR B-H: NA n = 5/group H: NA S-W: reported S-H: NA	2024	[64]
*Caralluma adscendens* R. Brown var. *bicolor*	AgNPs without an added delivery material	NA	In vitro scratch assay (L929 fibroblasts)	890.58 ± 0.05 µg/mL AgNPs, 24 h	PE	In vitro: ↑ L929 migration and scratch closure	NA	2024	[65]
*Aloe vera*	PVA nanofiber dressing containing AgNPs and *Ampelopsis brevipedunculata* extract	1 *w*/*w*	In vivo (rat burn open dorsal wound model)	NR	NC VC	In vivo: ↑ wound closure; ↓ inflammation and infection signs; ↑ re-epithelialization; ↑ collagen deposition; ↑ angiogenesis and mature vessels	R: NR B-W: NR B-H: NR n = 6 rats total ^8^ H: qual. S-W: reported S-H: NA	2024	[66]
*Camellia sinensis*	AgNPs-loaded topical chitosan gel	0.1% *w*/*w*	In vivo (*E. coli* and *S. aureus*-infected excision wound model in Wistar rats)	NR	NC VC AC (silver sulfadiazine cream 1%)	In vivo: ↑ infected wound contraction; ↑ re-epithelialization; ↑ epidermal regeneration; ↑ cell proliferation; ↑ organized collagen deposition	R: NR B-W: NR B-H: NR n = 6/group H: semi-quan. S-W: descriptive only S-H: descriptive only	2024	[67]
*Coriandrum sativum* L.	AgNPs without an added delivery material	NA	In vivo (*K. pneumoniae*-infected full-thickness wound model in BALB/c mice)	100 µL AgNP suspension ^9^, NR	NC CS-AgNP	In vivo: ↑ infected wound closure; ↓ bacterial burden; ↓ inflammation; ↑ collagen deposition; ↑ angiogenesis	R: reported B-W: NR B-H: NR n = NR H: qual. ^10^ S-W: reported S-H: NA	2024	[19]
*Petroselinum crispum*	AgNPs-loaded Vaseline ointment	1% *w*/*w*	In vivo (full-thickness burn wound model in Wistar rats)	Amount NR, once daily for 21 days	NC VC AC (silver sulfadiazine 1%)	In vivo: ↑ burn wound closure; ↑ re-epithelialization; ↑ granulation tissue formation; ↑ fibroblast activity; ↓ inflammatory cells; ↑ organized collagen and connective tissue	R: reported B-W: NR B-H: NR n = 6/group ^11^ H: qual. + quan. S-W: reported S-H: reported	2023	[68]
*Camellia sinensis*; *Ocimum sanctum*	AgNPs-loaded carrageenan-based dressing	NR	In vivo (*S. aureus*-infected full-thickness excision wound model in Swiss albino mice)	0.5 g dressing, every 3 days until healing	NC VC AgNP-only	In vivo: ↑ infected wound closure; ↑ re-epithelialization; ↑ dense collagen deposition; ↓ infection and inflammation	R: NR B-W: NR B-H: NA n = 4/group H: NA S-W: reported S-H: NA	2023	[69]
*Actinidia deliciosa*	AgNP–chitosan nanocomposite without a defined dosage form	NA	In vitro scratch assay (L929 fibroblasts)	12.5 µg AgNP nanocomposite, 24 h	NC PC (ascorbic acid)	In vitro: ↑ L929 fibroblast migration and scratch closure	NA	2023	[70]
*Potentilla fulgens*	AgNPs-loaded topical Carbopol gel	0.2% *w*/*w*	In vivo (excision wound model in Wistar rats)	NR	NC VC AC (marketed AgNO_3_ gel 0.2% *w*/*w*)	In vivo: ↑ wound contraction; ↑ re-epithelialization; ↑ collagen fiber formation; ↓ inflammatory cells	R: Reported B-W: NR B-H: NR n = 6 rats/group H: qual. S-W: reported S-H: NA	2023	[71]
*Camellia sinensis*; *Olea europaea* L.	AgNPs-loaded CMC hydrogel	0.01% *w*/*w*	In vitro scratch assay (3T3-L1 fibroblasts) Ex vivo diabetic foot ulcer wound exudate (myeloperoxidase, MPO, and collagenase inhibition)	In vitro: 100 µg/mL hydrogel, 24 h Ex vivo: 2 mg hydrogel, 30 min	NC	In vitro: ↑ scratch closure Ex vivo: ↓ MPO and collagenase activity	NA	2022	[72]
*Rhizophora apiculata*	AgNPs without an added delivery material	NA	In vitro scratch assay (L929 fibroblasts)	NR, 24 h	NC PE PC (ascorbic acid)	In vitro: ↑ L929 migration and scratch closure	NA	2022	[73]
*Scutellaria barbata*	AgNPs without an added delivery material	NA	In vitro scratch assay (L929 fibroblasts)	5 and 7.5 µg AgNPs, 24 h	NC	In vitro: ↑ L929 migration and scratch closure	NA	2021	[74]
*Gnaphalium polycaulon*	AgNPs without an added delivery material	NA	In vivo (excision wound model in male Wistar albino rats)	250 mg/kg body weight of AgNPs, NR	NC AC (Betadine ointment)	In vivo: ↑ wound closure; ↓ fibrosis; ↓ inflammatory infiltrate; ↑ angiogenesis	R: NR B-W: NR B-H: NR n = 6/group H: qual. S-W: NR S-H: NA	2021	[75]
*Echinophora platyloba* DC	Calendula flower oil–Vaseline ointment containing Chloroxine-conjugated AgNPs	NR	In vivo (excision wound model in male Wistar rats)	NR	NC VC	In vivo: ↑ wound closure; ↓ inflammation and swelling; ↑ re-epithelialization and skin regeneration	R: NR B-W: NR B-H: NR n = 6/group H: qual. S-W: reported S-H: NA	2021	[76]
*Tridax procumbens* L.	AgNPs-loaded topical chitosan gel	1% *w*/*w*	In vivo (full-thickness excision wound model in mice)	Amount NR, once daily until complete healing	NC AC (silver sulfadiazine 1% cream)	In vivo: ↑ wound closure; ↑ epithelialization; ↑ collagen deposition; ↑ skin regeneration; ↓ inflammation	R: NR B-W: NR B-H: NR n = 8/group H: qual. S-W: reported S-H: NA	2021	[77]
*Azadirachta indica*	AgNPs-loaded thermosensitive poloxamer hydrogel	0.3, 1 mg	In vivo (full-thickness excision wound model in male albino mice)	Hydrogels containing 0.3 or 1 mg AgNPs, NR ^12^	NC VC	In vivo: ↑ wound contraction; ↑ wound-healing efficacy; no skin irritation; 1 mg formulation showed the best healing response	R: NR B-W: NR B-H: NA n = 6/group H: NA S-W: reported S-H: NA	2021	[78]

^1^ Exact amount of AgNP/silver NR. ^2^ Extracts prepared by incubating 3 cm^2^ nanofiber and 0.5 cm^3^ hydrogel in DMEM for 2 weeks; 500 μL extract/well. ^3^ 2 wounds/animal. ^4^ Tested as AgNP gel alone, pre-irradiated AgNP gel, and post-application 660 nm-red-light irradiated AgNP gel. Pre-irradiated PP-AgNP gel showed best healing response. ^5^ Final hydrogel volume/mass and AgNP concentration NR. ^6^ Exact amount of AgNP/silver NR. ^7^ Exact amount of AgNP/silver NR. ^8^ 4 wounds/rat, per-group allocation NR. ^9^ Exact amount of AgNP/silver NR. ^10^ Immunohistochemistry quantification claimed but numerical data and statistical test NR. ^11^ 12 rats total, 2 wounds/rat, n = 6 wounds/group. ^12^ Applied once on day 1.

**Table 5:** Study-level evidence linking green synthesis process variables, AgNP properties, and therapeutic performance across the 34 included publications.

Relationship	Publications Directly Assessing the Relationship, n/34 (%)	Evidence Identified	Main Limitation	Refs.
Process → Property	14/34 (41.2%)	Seven publications used only UV–Vis/SPR outcomes, while seven also evaluated size, morphology, PDI, or zeta potential. One study used RSM; most others used OFAT or descriptive comparisons.	Replication and inferential analysis were often limited, and most optimization was based on optical responses rather than broader physicochemical characterization.	[48,50,51,52,53,54,57,58,61,64,67,68,71,76]
Property → Performance	0/34 (0%)	No direct relationship was identified.	Biological testing was generally performed only on the selected final batch. No study used property-controlled comparisons, correlation, regression, or mediation analysis to connect measured properties with performance.	—
Process → Performance	2/34 (5.9%)	Two publications compared therapeutic outcomes of AgNP systems prepared using different botanical sources.	Several material characteristics varied together, and the effect of the individual process variable could not be clearly isolated.	[54,58]
Complete Process → Property → Performance chain	0/34 (0%)	No publication directly connected a controlled process change to a measured property change and then showed that the property change accounted for therapeutic performance.	The three stages were usually evaluated separately rather than within one linked experimental design.	—

## Data Availability

No new data were created or analyzed in this study. Data sharing is not applicable to this article.

## Supplementary Materials

The following supporting information can be downloaded at: https://www.mdpi.com/article/10.3390/pharmaceutics18091161/s1, Table S1. Experimental design, analytical methods, and reported outcomes of release studies of plant-mediated silver nanoparticle formulations included in the review. Table S2. Reported stability and biodegradation assessments of plant-mediated silver nanoparticle dispersions and formulations included in the review. Table S3. Design and reported outcomes of safety-related assessments of plant-mediated silver nanoparticles and silver nanoparticle-containing wound-healing formulations included in the review.

## Abbreviations

The following abbreviations are used in this manuscript:

AgNPs	Silver nanoparticles
DLS	Dynamic light scattering
ECM	Extracellular matrix
FE-SEM	Field-emission scanning electron microscopy
FRAP	Ferric reducing antioxidant power
HA	Hyaluronic acid
HRMS	High-resolution tandem mass spectrometry
HUVECs	Human umbilical vein endothelial cells
MPO	Myeloperoxidase
MRSA	Methicillin-resistant *Staphylococcus aureus*
OFAT	One-factor-at-a-time
PDI	Polydispersity index
RSM	Response surface methodology
SEM	Scanning electron microscopy
SPR	Surface plasmon resonance
TEM	Transmission electron microscopy
TEWL	Transepidermal water loss
TFC	Total flavonoid content
TPC	Total phenolic content
UHPLC	Ultra-high-performance liquid chromatography
UV–Vis	Ultraviolet–visible spectroscopy
XRD	X-ray diffraction
ZIF-8	Zeolitic imidazolate framework-8
