# Supplementary material for: Plant-Mediated Silver Nanoparticle Formulations for Wound Healing: A Process–Property–Performance Perspective
Source: Pharmaceutics. 2026 Sep 15;18(9):1161. doi: 10.3390/pharmaceutics18091161 (PMC13610848; doi:10.3390/pharmaceutics18091161)
Supplement: Supplementary file 1 [file pharmaceutics-18-01161-s001.zip › pharmaceutics-4470380-supplementary.pdf]

Supplementary Materials

Plant-Mediated Silver Nanoparticle Formulations for Wound Healing: A Process–Property–Performance Perspective

Mohammad Moein Sadeghi [1,2], Souha Idoudi [1], Helena M. Kelly [2], Nashiru Billa [1,*]

[1] Department of Pharmaceutical Sciences, College of Pharmacy, QU Health, Qatar University, P.O. Box 2713, Doha, Qatar; m.moein@qu.edu.qa (M.M.S.); si1906739@qu.edu.qa (S.I.); nbilla@qu.edu.qa (N.B.)

[2] School of Pharmacy & Biomolecular Sciences, Royal College of Surgeons in Ireland (RCSI), D02 VN51 Dublin, Ireland; m.moein@qu.edu.qa (M.M.S.); helenakelly@rcsi.ie (H.M.K.)

* Correspondence: nbilla@qu.edu.qa

Table S1. Experimental design, analytical methods, and reported outcomes of release studies of plant-mediated silver nanoparticle formulations included in the review.

Abbreviations: CMC, carboxymethyl cellulose; Da, dalton; kDa, kilodalton; HAase, hyaluronidase; ICP–OES, inductively coupled plasma optical emission spectrometry; M, molar; NR, not reported

Study	Material tested	Comparator material(s)	Released species	Release medium and conditions	Study duration and sampling times	Analytical method	Release results	Ref.
Shivam et al., 2026	*Hibiscus rosa-sinensis/Cymbopogon citratus*-mediated AgNPs-loaded topical Carbopol gel	Marketed colloidal-silver gel (Megaheal®)	AgNPs[1]	5 mL gel in a 14,000-Da dialysis bag; 250 mL phosphate buffer, pH 7.2; 37 +/- 0.5 degrees C; 100 rpm; maintained sink conditions	8 h; 0, 15, 30, 60, 120, 180, 240, and 480 min	Aliquots quantified by UV-Vis at 417 nm	~27% burst release at 15 min<br><br>88.9% release at 8 h vs 68.9% from marketed gel	[1]
Wang et al., 2025	pH and hyaluronidase-responsive hyaluronic acid hydrogel containing *Aronia melanocarpa*-mediated AgNPs	No comparator materials	AgNPs[2]	pH response: PBS at pH 5.5, 6.4, or 7.4; maintained sink conditions<br><br>HAase response: PBS pH 7.4 containing 0, 0.2, 0.5, 1.0, or 2.0 micrograms/mL HAase; maintained sink conditions<br><br>Dual response: 2.0 micrograms/mL HAase + pH 6.4; maintained sink conditions<br><br>Hydrogel amount NR	pH study: 48 h; time points NR<br><br>HAase study: single 30-min incubation	UV-Vis absorbance, wavelength NR	24-h release: 91.3% at pH 5.5 and 89.6% at pH 6.4; lower at pH 7.4<br><br>HAase increased release dose-dependently; pH 6.4 added 12.6% vs HAase alone	[2]
Chang et al., 2025	Citrus pectin hydrogel loaded with *Citrus aurantium*-mediated AgNPs	No comparator materials	Ag^+	PBS at pH 7.4 or 5.4<br>0.5 mL; maintained sink conditions<br><br>Hydrogel amount NR	24 h; 0, 2, 4, 8, 12, and 24 h	Ag+ in supernatant quantified by ICP-OES	Gradual silver release at both pH values, reaching approximately 2.3 ppm at pH 7.4 and 2.1 ppm at pH 5.4 by 24 h	[3]
Ruffo et al., 2022	CMC hydrogel loaded with *Camellia sinensis/Olea*	No comparator materials	AgNPs[3]	50 mg hydrogel in 6 mL 0.1 M PBS, pH 8, inside a 12-14 kDa dialysis bag; immersed in 40 mL	8h; 1, 2, 4, 6, and 8 h	UV-Vis absorbance at 430 nm	Burst release reached 71.5 +/- 0.5% by 2 h, followed by	[4]

[1] Reported as AgNPs; intact AgNPs, Ag^+, and total silver were not distinguished.
[2] Reported as AgNPs; intact AgNPs, Ag^+, and total silver were not distinguished.
[3] Reported as AgNPs; intact AgNPs, Ag^+, and total silver were not distinguished.

	europaea-mediated AgNPs			PBS at 37 +/- 0.5 degrees C under constant agitation			slower release to 90 +/- 0.1% by 8 h	

Table S2. Reported stability and biodegradation assessments of plant-mediated silver nanoparticle dispersions and formulations included in the review.

Abbreviations: Alg, alginate; DLS, dynamic light scattering; DMEM, Dulbecco's modified Eagle medium; mM, millimolar; NR, not reported; PDI, polydispersity index; RH, relative humidity; SPR, surface plasmon resonance

**Study**	**Material tested**	**Comparator material(s)**	**Stability domain**	**Storage/test conditions**	**Study duration; time points**	**Stability indicators and methods**	**Stability results**	**Ref.**
Tahamtan et al., 2026	PVA-Alg nanofiber containing *Pinus eldarica*-mediated AgNPs	AgNP-free PVA-Alg scaffold	In-use/formulation biodegradation	Immersion in 10 mL PBS, pH 7.4, at 37°C under gentle agitation	14 days; days 3, 7, and 14	Gravimetric weight-loss assessment	Lower weight loss versus PVA–Alg<br><br>Slower degradation (statistical significance NR)	[5]
Shivam et al., 2026	*H. rosa-sinensis/C. citratus*-mediated AgNPs-loaded topical Carbopol gel	No comparator materials	Storage/formulation physical stability	2-8 degrees C<br><br>30 +/- 2 degrees C/65% RH<br><br>40 +/- 2 degrees C/75% RH	3 months; day 0, months 1, 2, and 3	Visual appearance, pH, texture, leakage, and phase separation	Appearance/texture unchanged; pH 6.0–6.5<br><br>Leakage/phase separation absent at baseline; later NR	[1]
Ahmed et al., 2026	*Punica granatum*-mediated AgNP aqueous dispersion without an added delivery material	No comparator materials	Colloidal/ AgNP optical stability	NR	~ 64 days; 2, 24, 48, 96, 120, 144, 168, 192, 264, 408, 936, and 1536 h	UV-Vis spectroscopy (300-1000 nm): qualitative SPR peak position, shape, intensity, and broadening	SPR position/shape broadly stable to 1536 h, but absorbance decreased	[6]
Sharma et al., 2025	Carbopol hydrogel containing *Cyperus rotundus*-mediated AgNPs	No comparator materials	Short-term Storage/formulation physical stability	Stored in containers under refrigeration, then containers were inverted	Overnight; NR	Visual observation for phase separation	No phase separation observed after overnight refrigeration	[7]
Huang et al., 2024	Coaxial PVA nanofiber dressing containing *Aloe vera*-mediated AgNPs and *Ampelopsis brevipedunculata* extract	Electrosprayed PVA nanofiber dressing containing *A. vera*-mediated AgNPs and *A. brevipedunculata* extract	In-use/formulation biodegradation	PBS immersion	14 days; NR	Gravimetric weight loss	Weight loss plateaued at approximately 45% for PVA, 40% for electrosprayed fiber, and 33% for coaxial fiber<br><br>Coaxial fiber degraded more slowly than the electrosprayed formulation	[8]
Bharali et al., 2023	Selected *Potentilla fulgens*-mediated AgNP dispersion (prepared with 5 mg root extract and 3 mM $AgNO_3$)	Five other candidate AgNP dispersions prepared with different	Colloidal/ AgNP optical stability	NR	1 month; 24 h, 1 week, 3 weeks, and 1 month	Qualitative UV-Vis peak rating/sharpness; DLS particle size and PDI	The selected dispersion remained stable for 1 month (PDI 0.20–0.22; size 137–153 nm; zeta potential −25.5 mV)	[9]

		extract/AgNO3 combinations						
Fatima et al., 2021	Chitosan topical gel loaded with *Tridax procumbens*-mediated AgNPs	No comparator materials	Short-term physical stress	2-day freeze-thaw cycles (45 degrees C and 4 degrees C); separate sample centrifuged at 3000 rpm for 30 min at 6 degrees C	2 days; day 2	Visual inspection for phase separation and sedimentation	No phase separation or sedimentation was observed	[10]
Chinnasamy et al., 2021	*Azadirachta indica*-mediated AgNPs without an added delivery material	No comparator materials	Colloidal/ AgNP optical stability	AgNPs dispersed separately in distilled water, PBS (pH 7.4), 0.9% NaCl, DMEM, and complete medium	28 days; days 1, 7, 14, 21, and 28	UV-Vis absorbance from 100-900 nm	The ~400 nm SPR band persisted across all media and for 28 days in water, with some absorbance reduction	[11]

Table S3. Design and reported outcomes of safety-related assessments of plant-mediated silver nanoparticles and silver nanoparticle-containing wound-healing formulations included in the review.

Control labels: AC, active comparator; CS–AgNP, chemically synthesized silver nanoparticle; AgNP-only, AgNPs tested without the accompanying delivery formulation; NC, negative control; PC, positive control; PE, plant-extract control; VC, vehicle or AgNP-free formulation control.

Other abbreviations: NA, not applicable; NR, not reported; ADMET, absorption, distribution, metabolism, excretion, and toxicity; ALT, alanine aminotransferase; AO–EB, acridine orange–ethidium bromide; AST, aspartate aminotransferase; CBC, complete blood count; CCK-8, Cell Counting Kit-8; F1, first filial generation; h-CLAT, human Cell Line Activation Test; OECD, Organisation for Economic Co-operation and Development; PLT, platelets; ProTox-II, web-based toxicity-prediction platform; STZ, streptozotocin; TOPKAT, Toxicity Prediction by Komputer Assisted Technology

Study	Material tested	Safety domain	Model	Assay/ endpoints	Administered dose	Exposure duration	Route	Control and comparator group(s)	Main safety result	Ref.
Yu et al., 2026	*Pyrrosia petiolosa*-mediated AgNPs without an added delivery material	Cytocompatibility	Primary mouse lymphocytes	MTT	NR	44 h	In vitro exposure	NC<br>CS-AgNP	Lower cytotoxicity than CS-AgNP	[12]
Tahamtan et al., 2026	PVA-Alg nanofiber containing *P. eldarica*-mediated AgNPs	Hemocompatibility	Rat whole blood	Hemoglobin release	NR	60 min	In vitro direct blood contact	NC<br>PC (distilled water)<br>VC	Below 5% hemolysis threshold	[5]
		Cytocompatibility	L929 fibroblasts	MTT cell-viability assay	NR[4]	1, 3, and 5 days	In vitro exposure		Cytocompatible at 32-128 µg/mL<br><br>Reduced viability at 256 µg/mL	
Karthikeyan et al., 2026	*Euphorbia hirta/ Ocimum americanum* -mediated AgNPs without an added delivery material	Embryotoxicity	*Zebrafish (Danio rerio)* embryos	Hatching rate; survival rate; developmental deformities	5, 10, 20, 40, and 80 µg/mL[5]	78 h post-fertilization; endpoints monitored every 24 h for 3 days	Aqueous embryonic exposure	NC	Dose-dependent embryotoxicity at 40-80 µg/mL	[13]

[4] 32, 64, 128, and 256 µg/mL as reported loading doses; exact AgNP/silver concentration NR
[5] Exact AgNP/silver concentration NR

Shivam et al., 2026	Developed HC-CC AgNP Carbopol gel (described inconsistently as HC-AgNPs/AgNPs in Results/Fig. 5B)	Acute irritation/vascular compatibility	Hen's egg test chorioallantoic membrane (HET-CAM)	Visible discoloration, bleeding, clotting, or lysis; irritation score	0.2 mL of 0.2% w/v gel[6]	Observed to 5 min	Direct application onto CAM	PC (NaOH)	No visible lysis or bleeding<br><br>Irritation score = 0	[1]
Wang et al., 2025	pH and hyaluronidase-responsive hyaluronic acid hydrogel containing *Aronia melanocarpa*-mediated AgNPs	Cytocompatibility	L929 fibroblasts	CCK-8 cell viability	NR	24 h	In vitro exposure	NC<br>VC	Viability remained >80% at all concentrations (lowest: ~85% at nominal 30 mM $AgNO_3$).	[2]
		Hemocompatibility	NR	Quantitative hemolysis ratio	NR	NR	In vitro blood contact	VC<br>AC (medical Ag^+ wound dressing)	<5% at nominal $AgNO_3$ levels of 0–20 mM<br><br>30 mM caused ~6% hemolysis	
		In vivo systemic safety	full-thickness MRSA-infected wound model in rats	H&E histology of heart, liver, spleen, lung, and kidney; serial body weight	NR	12 days	Topical treatment	NC<br>VC<br>AC (medical Ag^+ wound dressing)	No organ toxicity or abnormal weight changes at day 12	
Sharma et al., 2025	Carbopol hydrogel containing *C. rotundus*-mediated AgNPs	Cytocompatibility	HEK293 embryonic kidney cells	MTT	200, 400, 600, 800, and 1000 µg/mL hydrogel[7]	72 h	In vitro exposure	NC<br>VC	At 1000 µg/mL after 72 h, viability was 98% for free AgNPs and 92% for AgNP hydrogel	[7]
		In vivo dermal toxicity	NR	Modified OECD 404 skin-tolerance observation	0.5 g hydrogel	NR	Topical application	NC	No reaction or redness through day 15	
Palanisamy et al., 2025	*Nigella sativa*-mediated AgNPs without an added delivery material	Cytocompatibility	HaCaT keratinocytes	MTT	6.25, 12.5, 25, 50, and 100 µg/mL	24 h	In vitro exposure	NC<br>PE	24-h metabolic activity remained ≥~100%<br><br>At 100 µg/mL: ~156% for AgNPs vs ~124% for plant extract	[14]
Guzzatti et al., 2025	*Curcuma longa*-mediated AgNP suspension	Cytocompatibility	NIH-3T3 fibroblasts	MTT	1%, 5%, 10%, and 20% of 0.125	24 h	In vitro exposure	NC	Viability decreased significantly at 10–20%, reaching ~80% at 20%	[15]

[6] Exact AgNP/silver concentration NR
[7] Exact AgNP/silver concentration NR

	Euterpe oleracea-mediated AgNP suspension				mg/mL hydrogel				Viability decreased significantly at 5–20%, with a minimum of ~82%	
Govindarasu et al., 2025	Chitosan-encapsulated *Lannea coromandelica*-mediated AgNP system without a defined dosage form	Cytocompatibility	NIH-3T3 fibroblasts	MTT	25 µg/mL	24 and 48 h	In vitro exposure	NC<br>CS-AgNP	Viability was approximately 82% and 90% vs 62% and 64% with chemically synthesized AgNPs at 24 and 48 h, respectively.	[16]
Erol et al., 2025	Chitosan–modified styrenic polymer nanocomposite containing dispersed *Prunus spinosa*-mediated AgNPs; no defined dosage form	Cytocompatibility	L929 fibroblasts	MTT	10, 25, 50, 100, 250, 500, and 1000 µg/mL	24 h	In vitro exposure	NC<br>VC<br>AgNP-only	All nanocomposites remained non-cytotoxic at 50 µg/mL; viability declined at higher doses.<br><br>Free AgNPs were more cytotoxic	[17]
Chang et al., 2025	Citrus pectin hydrogel loaded with *C. aurantium*-mediated AgNPs	Cytocompatibility	HUVECs and NIH-3T3 fibroblasts	MTT	NR	24 h	In vitro exposure	NC<br>PE<br>VC<br>AgNP-only	No cytotoxicity; AgNP hydrogels showed higher metabolic activity than free AgNPs	[3]
		Hemocompatibility	Mouse RBCs and platelets	Hemolysis ratio, RBC morphology, and platelet aggregation/turbidity	NR	4 h	In vitro blood contact	NC<br>PE<br>VC<br>AgNP-only<br>PC (ddH_2O; PBS + thrombin)	Negligible hemolysis and platelet aggregation; RBC morphology preserved	
		Short-term in vivo systemic safety	Male STZ-diabetic C57BL/6 mice; n = 5 for body weight; terminal-test n NR	Body weight, CBC, ALT/AST, urea/creatinine, and major-organ H&E	100 µL of 50 µg/mL AgNP hydrogel	Applications on days 1, 3, 5, 7, and 9	Topical application	NC<br>PE<br>VC<br>AgNP-only	Stable body weight, normal blood/biochemical markers, and no major-organ lesions	
Verma et al., 2024	Thermosensitive poloxamer hydrogel loaded with *Premna*	Acute in vivo dermal toxicity	Unwounded Swiss albino mice	Inflammation, irritation/swelling, edema, clinical	NR	Single application [8]	Topical application	VC (extract-loaded, AgNP-free hydrogel)	No inflammation/edema at 24 h or	[18]

[8] Contact duration NR; observations to 24 h and day 14

	integrifolia-mediated AgNPs (10% w/w)			toxicity, and mortality					toxicity/death through day 14.	
Murugapandi et al., 2024	*Caralluma adscendens*-mediated AgNPs without an added delivery material	Cytocompatibility	L929 fibroblasts	MTT, AO-EB apoptosis staining	0-1000 µL	24 h	In vitro exposure	NC<br>PE	Dose-dependent viability loss: about 42% at the highest reported level	[19]
Huang et al., 2024	Coaxial PVA nanofiber dressing containing *A. vera*-mediated AgNPs and *A. brevipedunculata* extract	Cytocompatibility	L929 fibroblasts	MTT	NR	24 and 48 h	Direct culture on fiber	VC<br>AC (electrosprayed fiber)	Both AgNP-containing formulations had higher OD than PVA at 24 and 48 h	[8]
Ahmad B. et al., 2024	*Coriandrum sativum*-mediated AgNPs without an added delivery material	Cytocompatibility	HUVEC endothelial cells	MTT	0, 10, 20, 30, 40, 50, and 60 µg/mL	24 h	In vitro exposure	NC<br>CS-AgNP	Viability remained ≥~75% up to 60 µg/mL<br><br>Responses were broadly similar between green and chemical AgNPs.	[20]
		Hemocompatibility	Fresh mouse RBCs	Hemolysis and erythrocyte morphology	2, 4, 6, 8, and 10 µg/mL	3 h	Direct ex vivo blood exposure	NC<br>PC (deionized water)<br>CS-AgNP	Green AgNPs caused less hemolysis, but exceeded 5% at ≥4 µg/mL (max 11.18%)<br><br>CS-AgNPs caused 16.99–42.16%.	
		Short-term in vivo systemic safety	Female BALB/c mice with infected open wounds	Body weight; WBC, RBC, HGB, PLT; AST, ALT, urea, creatinine; H&E of heart, liver, lung, spleen, and kidney	100 µL[9]	NR	Topical application	NC<br>CS-AgNP	No systemic toxicity was detected at day 12	
Singh et al., 2023	*Camellia sinensis/Ocimum sanctum*-mediated	In silico ADMET/toxicity prediction	pkCSM, SwissADME, TOPKAT,	Predicted absorption, distribution, metabolism,	NA	NA	NA	AC (ciprofloxacin)	AgNPs and kappa-carrageenan were assigned predicted toxicity class 3	[21]

[9] AgNP concentration/mass NR

	AgNPs and kappa-carrageenan were evaluated separately		and ProTox-II	excretion, organ toxicity, Tox21 pathways, LD50, and toxicity class					(predicted LD50 100 and 225 mg/kg, respectively), versus class 4 for ciprofloxacin	
Kodasi et al., 2023	*Actinidia deliciosa*-mediated AgNP–chitosan nanocomposite without a defined dosage form	Cytocompatibility	L929 fibroblasts	MTT	20, 40, 60, 80, and 100 µg/mL	24 h	In vitro exposure	NC<br>PC (cisplatin)	L929 viability decreased dose-dependently from 86.68% to 42.87% (20–100 µg/mL; IC_{50}: 83.52 µg/mL), with morphological changes at higher doses.	[22]
Bharali et al., 2023	Topical Carbopol gel loaded with *P. fulgens*-mediated AgNP	Acute in vivo dermal toxicity	Unwounded rats	Mortality; skin irritation, inflammation, and swelling; behavioral changes	Single 200 mg/kg body-weight dose	24 h	Topical application to shaved back	NR	No mortality, skin irritation, inflammation, swelling, or behavioral changes were reported over 14 days.	[9]
Ruffo et al., 2022	CMC hydrogel loaded with *C. sinensis/O. europaea*-mediated AgNPs	Cytocompatibility	3T3-L1 fibroblasts	MTT	45-100 µg/mL	24 h	In vitro exposure	NC	Viability remained 88-97% across the tested concentrations, with no decrease reaching 20%	[4]
		In vitro skin sensitization	THP-1 monocytic leukemia cells	OECD 442E h-CLAT: PI viability/CV75 and CD54/CD86 relative fluorescence	7.8, 22.7, 55.3, 112.3, 217.3, 514.3, 775.2, and 1112.2 µg/mL	24 h	In vitro exposure	NC<br>PC (2,4-dinitrochlorobenzene, DNCB)	CD54/CD86 responses were interpreted as below the sensitization criteria	
		In vitro skin irritation	Reconstructed human epidermis (EPISKIN, EpiDerm)	OECD 439 MTT tissue viability; n=3	25 mg	60 min	Topical in vitro exposure	NC<br>PC (sodium dodecyl sulfate, SDS)	Reconstructed-epidermis viability remained above 50%	
Alsareii et al., 2022	*Rhizophora apiculata* -mediated AgNPs without an added delivery material	Cytocompatibility	L929 fibroblasts	MTT	10, 20, 30, 40, and 50 µg/mL	NR	In vitro exposure	NC<br>PE	AgNPs were less cytotoxic than PE (77.5% vs. 46.8% viability at 50 µg/mL; IC_{50}: 105.5 vs. 47.47 µg/mL)	[23]
Veeraraghavan et al., 2021	*Scutellaria barbata*-mediated	Cytocompatibility	L929 fibroblasts	MTT	2.5, 5, 7.5, 10, and 15 µg/mL	24 h	In vitro exposure	NC	Viability remained above approximately	[24]

	AgNPs without an added delivery material								90% through 15 µg/mL over 24 h	
Shanmugapriya et al., 2021	*Gnaphalium polycaulon*-mediated AgNPs without an added delivery material	Local wound-site tolerability	Rats with dorsal excision wounds	Visual wound observation and qualitative H&E	250 mg/kg body weight	NR	Application to the open wound	NC<br>AC (Betadine ointment)	No visible suppuration occurred, but H&E showed a foreign-body giant-cell reaction	[25]
Shahabadi et al., 2021	Calendula flower oil–Vaseline ointment containing Chloroxine-conjugated AgNPs synthesized by *Echinophora platyloba*	In vivo tolerability and limited hematology	rats with excision wounds	Mortality/growth observations; WBC, RBC, hemoglobin, and platelets	NR	NR	Topical application to the open wound	NC<br>VC	No mortality or growth abnormalities occurred; ointment B increased WBC and platelets and reduced RBC	[26]
Fatima et al., 2021	Chitosan topical gel loaded with *T. procumbens*-mediated AgNPs	Hemocompatibility	EDTA-treated erythrocytes	Hemoglobin-release	NR	1 h	Direct erythrocyte exposure	NC<br>AgNP-only<br>VC<br>PC (sodium dodecyl sulfate)	Hemolysis was 32.87% for AgNP-only, 5.67% for vehicle, and 16.88% for AgNP-loaded gel. Formulation reduced hemolysis relative to AgNP-only.	[10]
Chinnasamy et al., 2021	*A. indica*-mediated AgNPs without an added delivery material	In vivo developmental and reproductive toxicity	Drosophila melanogaster; 50 eggs or 50 females	Adult eclosion; egg laying; F1 eclosion; larval-midgut TEM	10, 25, 50, 100, and 250 µg/mL in food	10-day[10]	Dietary	NC<br>PE	Adult eclosion was unaffected up to 250 µg/mL; reproductive effects appeared at 250 µg/mL. AgNP intestinal uptake occurred without visible phenotypic damage.	[11]
	Poloxamer hydrogel loaded with *A. indica*-	Acute dermal irritation	Healthy adult mice	Visual skin redness, dryness, and flakiness	20 µL of 3-mg-labelled AgNP hydrogel	NR[11]	Topical application	VC	No visible skin irritation occurred within 48 h	

[10] Egg-to-adult eclosion; 10-day exposure for egg laying; parental exposure for 10, 20, or 30 days before F1 assessment
[11] Applied once on day 1

	mediated AgNPs									

References:

1. Gupta, T.; Badhwar, R.; Singh, R. Green synthesis of Carbopol-silver nanoparticles using *Hibiscus rosasinensis* and *Cymbopogon citratus*: A synergistic in vitro and in vivo approach for accelerated wound healing in Swiss albino mice. *Biocatal. Agric. Biotechnol.* **2026**, *73*.
2. Wang, X.; Huang, J.; Zhao, J.; Peng, L.; Qi, L.; Wu, T.; He, J.; Gao, Y.; Zhou, Y. Dual stimuli-responsive and self-healing hyaluronic acid-based nanocomposite hydrogels for promoting wound healing of methicillin-resistant Staphylococcus aureus infection. *Int J Biol Macromol* **2025**, *320*, 145829.
3. Chang, L.; Ahmad, B.; Tong, A.; Wu, Z.; Xu, P.; Gao, Z.; Yin, C.; Luan, M.; Tong, C.; Liu, B. Green synthesis of silver nanoparticles using Fructus Aurantii-loaded Citrus pectin hydrogel for accelerated healing of MRSA-infected diabetic wounds. *Int J Biol Macromol* **2025**, *315*, 144222.
4. Ruffo, M.; Parisi, O.I.; Dattilo, M.; Patitucci, F.; Malivindi, R.; Pezzi, V.; Tzanov, T.; Puoci, F. Synthesis and evaluation of wound healing properties of hydro-diab hydrogel loaded with green-synthetized AGNPS: in vitro and in ex vivo studies. *Drug Deliv Transl Res* **2022**, *12*, 1881-1894.
5. Tahamtan, M.S.; Ansari, F.; Moharrami Kasmaie, F.; Zareshahrabadi, Z.; Amani, A.M.; Salehi, M.; Amiri, S.; Ghasemian, S.; Askari Jahromi, M.; Vaez, A. Integration of green synthesized silver nanoparticles in electrospun PVA-alginate and resveratrol-loaded ZIF-8 into alginate lyophilized hydrogel as a dual-layered biomimetic skin construct. *Int J Biol Macromol* **2026**, *338*, 149681.
6. Ahmed, R.S.; Mohammed, I.R.; Ajeel, A.K. In Vivo Wound Healing Activity Using Pomegranate Peel Derived – Silver Nanoparticle Hydrogel Pre-Exposed with 660 nm Red Light. *Biol. Trace Elem. Res.* **2026**.
7. Sharma, S.; Bose, A.; Biswas, S.; Sen, S.; Roy, I. Cyperus rotundus mediated green synthesis of silver nanoparticles for antibacterial wound dressing applications. *Sci Rep* **2025**, *15*, 18394.
8. Huang, T.Y.; Lin, J.Y.; Su, W.T. Coaxial nanofibers encapsulated with Ampelopsis brevipedunculata extract and green synthesized AgNPs for wound repair. *Colloids Surf B Biointerfaces* **2024**, *235*, 113771.
9. Bharali, A.; Sarma, H.; Biswas, N.; Kalita, J.M.; Das, B.; Sahu, B.P.; Prasad, S.K.; Laloo, D. Green synthesis of silver nanoparticles using hydroalcoholic root extract of *Potentilla fulgens* and evaluation of its cutaneous wound healing potential. *Mater. Today Commun.* **2023**, *35*.
10. Fatima, F.; Aldawsari, M.F.; Ahmed, M.M.; Anwer, M.K.; Naz, M.; Ansari, M.J.; Hamad, A.M.; Zafar, A.; Jafar, M. Green Synthesized Silver Nanoparticles Using *Tridax Procumbens* for Topical Application: Excision Wound Model and Histopathological Studies. *Pharmaceutics* **2021**, *13*.
11. Chinnasamy, G.; Chandrasekharan, S.; Koh, T.W.; Bhatnagar, S. Synthesis, Characterization, Antibacterial and Wound Healing Efficacy of Silver Nanoparticles From Azadirachta indica. *Front Microbiol* **2021**, *12*, 611560.
12. Yu, Z.; Xie, C.; Liu, M.; Chen, Y.; Liu, H.; Zhang, J.; Wang, Q.; Lei, P.; Yan, F.; Long, Y. Eco-Synthesized silver nanoparticles from Pyrrosia petiolosa: lymphocyte-sparing activity and targeted full-Thickness traumatic wound healing via motor protein modulation. *Green Chemistry Letters and Reviews* **2026**, *19*.
13. Karthikeyan, E.; Rajeshkumar, S.; Nandhini, J. Microwave-Assisted Green Synthesis of Silver Nanoparticles Using Euphorbia hirta and Ocimum americanum: Optimization, Characterization, and Biomedical Applications. *Biomedical Mater. Devices* **2026**, *4*, 2230-2249.
14. Palanisamy, C.P.; Poompradub, S.; Sansanaphongpricha, K.; Jayaraman, S.; Subramani, K.; Sonsudin, F. Green synthesis of Nigella sativa-mediated silver nanoparticles for enhanced antibacterial activity and wound healing: Mechanistic insights and biomedical applications. *Environmental Nanotechnology, Monitoring & Management* **2025**, *24*.
15. Guzzatti, M.F.M.; de Moura, A.B.; Venturini, L.M.; de Roch Casagrande, L.; Lima, I.R.; da Costa, C.; de Pieri, E.; Tietbohl, L.T.W.; Feuser, P.E.; Machado-de-Ávila, R.A.; et al. Biosynthesized Silver Nanoparticles Modulate Inflammation in a Palatine Wound Model. *Clin Exp Dent Res* **2025**, *11*, e70213.
16. Govindarasu, M.; Vaiyapuri, M.; Thiruvengadam, M. Green synthesis of chitosan–silver nanoparticles using Lannea coromandelica: A synergistic in vitro and in vivo approach for accelerated wound healing in BALB/c mice. *Biocatal. Agric. Biotechnol.* **2025**, *70*.
17. Erol, I.; Hazman, Ö.; Khamidov, G.; Büyükben, A. Enhancement of thermal and biological properties of chitosan by a styrenic polymer and biosynthesized silver nanoparticles. *Mater. Today Commun.* **2025**, *44*.
18. Verma, P.; Dixit, J.; Singh, C.; Singh, A.N.; Singh, A.; Tiwari, K.N.; Muthu, M.S.; Nath, G.; Mishra, S.K. Preparation of hydrogel from the hydroalcoholic root extract of Premna integrifolia L. and its mediated green synthesis of silver nanoparticles for wound healing efficacy. *Mater. Today Commun.* **2024**, *41*.

19. Murugapandi, M.; Elanchezhiyan, S.S.; Oh, T.H.; Ramasundaram, S.; Muniyappan, N. An evaluation of the pharmacological responses of metal nanoparticles derived from aqueous extract of *Caralluma adscendens* R. Brown var. *bicolor*. *J Mol Liq* **2024**, *401*.
20. Ahmad, B.; Chang, L.; Yin, C.; Wu, Z.; Tong, A.; Tong, C.; Liu, B. Assessing Phytogenic and Chemogenic Silver Nanoparticles for Antibacterial Activity and Expedited Wound Recuperation. *Nanomaterials (Basel)* **2024**, *14*.
21. Singh, K.; Yadav, V.B.; Yadav, U.; Nath, G.; Srivastava, A.; Zamboni, P.; Kerkar, P.; Saxena, P.S.; Singh, A.V. Evaluation of biogenic nanosilver-acticoat for wound healing: A tri-modal in silico, in vitro and in vivo study. *Colloids and Surfaces a-Physicochemical and Engineering Aspects* **2023**, *670*.
22. Kodasi, B.; Kamble, R.R.; Shettar, A.K.; Hoskeri, J.H.; Keri, R.S.; Metre, T.V.; Bheemayya, L.; Nadoni, V.B.; Nayak, M.R. Novel jointured green synthesis of chitosan-silver nanocomposite: An approach towards reduction of nitroarenes, anti-proliferative, wound healing and antioxidant applications. *Int J Biol Macromol* **2023**, *246*, 125578.
23. Alsareii, S.A.; Manaa Alamri, A.; AlAsmari, M.Y.; Bawahab, M.A.; Mahnashi, M.H.; Shaikh, I.A.; Shettar, A.K.; Hoskeri, J.H.; Kumbar, V. Synthesis and Characterization of Silver Nanoparticles from Rhizophora apiculata and Studies on Their Wound Healing, Antioxidant, Anti-Inflammatory, and Cytotoxic Activity. *Molecules* **2022**, *27*.
24. Veeraraghavan, V.P.; Periadurai, N.D.; Karunakaran, T.; Hussain, S.; Surapaneni, K.M.; Jiao, X. Green synthesis of silver nanoparticles from aqueous extract of Scutellaria barbata and coating on the cotton fabric for antimicrobial applications and wound healing activity in fibroblast cells (L929). *Saudi J Biol Sci* **2021**, *28*, 3633-3640.
25. Shanmugapriya, K.; Palanisamy, S.; Boomi, P.; Subaskumar, R.; Ravikumar, S.; Thayumanavan, T. An eco-friendly Gnaphalium polycaulon mediated silver nanoparticles: Synthesis, characterization, antimicrobial, wound healing and drug release studies. *J. Drug Deliv. Sci. Technol.* **2021**, *61*.
26. Shahabadi, N.; Zendehcheshm, S.; Khademi, F.; Rashidi, K.; Chehri, K.; Dehpahni, M.F. Green synthesis of Chloroxine-conjugated silver nanoflowers: Promising antimicrobial activity and in vivo cutaneous wound healing effects. *J. Environ. Chem. Eng.* **2021**, *9*.
